# Spatial single-cell landscape of tumor-associated macrophages and their crosstalk with the tumor microenvironment

**DOI:** 10.1038/s41421-026-00888-3

**Published:** 2026-05-19

**Authors:** Rui-chao Nie, Guo-sheng Hu, Shi-qiang Cao, An Wang, Du-chuang Wang, Wen Liu

**Affiliations:** 1 National Institute for Data Science in Health and Medicine, Xiamen University, Xiamen, Fujian, China; 2 State Key Laboratory of Vaccines for Infectious Diseases, Xiang An Biomedicine Laboratory, School of Pharmaceutical Sciences, Faculty of Medicine and Life Sciences, Xiamen University, Xiamen, Fujian, China; 3 Fujian Provincial Key Laboratory of Innovative Drug Target Research, School of Pharmaceutical Sciences, Faculty of Medicine and Life Sciences, Xiamen University, Xiamen, Fujian, China; 4 Biomedical Research Center of South China, College of Life Sciences, Fujian Normal University, Fuzhou, Fujian, China; 5 Department of Thoracic Surgery, Fujian Medical University Union Hospital, Fuzhou, Fujian, China; 6 Shenzhen Research Institute of Xiamen University, Shenzhen, China

**Keywords:** Cancer microenvironment, Immunoediting

## Abstract

Tumor-associated macrophages (TAMs) constitute a critical immune cell population within the tumor microenvironment (TME) and exhibit high functional heterogeneity. In this study, a pan-cancer atlas comprising 28 TAM subtypes was systematically constructed by integrating single-cell transcriptomic and spatial transcriptomic data from 291 human samples across 16 cancer types. The biological characteristics and spatial distribution patterns of these subtypes within the TME as well as the interaction mechanisms between TAMs and TME components closely associated with tumor progression were elucidated. TAMs localized in the peritumoral or core regions of tumors participate in angiogenesis and metabolic reprogramming, promoting further tumor development. The retention of CD8^+^ T cells by TAMs can induce local inflammation and an immunosuppressive microenvironment. Additionally, cancer-associated fibroblasts (CAFs) play crucial roles in the polarization and survival of TAMs and are involved in their recruitment and activation. TAMs facilitate tumor invasion, metastasis and immune evasion through secreted phosphoprotein 1 (SPP1) and interaction with the integrin family/CD44 axis in CAFs. This study deepens the understanding of the biological characteristics of TAMs and provides new theoretical foundations and potential targets for cancer treatment strategies targeting specific TAM subpopulations.

## Introduction

The tumor microenvironment (TME) is a vast ecosystem composed of various cellular components, including tumor cells, stromal cells, and immune cells. These components engage in complex interactions with one another^1^. Tumor-associated macrophages (TAMs) are highly diverse and crucial components of the TME and extensively participate in a multitude of biological functions, such as phagocytosis, antigen presentation, inflammation, angiogenesis, and immune evasion^2–4^. TAMs have been reported to be associated with a poor prognosis in cancer patients. Traditionally, macrophages are classified into “classically activated” (M1) and “alternatively activated” (M2) subtypes. M1 and M2 subtypes are present throughout all stages of tumor development and are often involved in a dynamic process of interconversion^5^. However, this subtyping approach is characterized by ambiguous boundaries and inherent limitations. Currently, research on TAMs focuses primarily on their intrinsic characteristics, while the crosstalk between TAMs and other TME components is often overlooked. However, these complex signaling processes may represent critical avenues for identifying key therapeutic strategies for tumors. For example, recent studies have demonstrated that interactions between CAFs and SPP1⁺ TAMs, as well as functional state switching of TAMs, play critical roles in tumor progression and the response to immunotherapy across multiple cancer types. These processes contribute to stromal remodeling and the establishment of an immunosuppressive microenvironment, and TAM polarization states and plasticity are now recognized as key determinants influencing the efficacy of immune checkpoint blockade therapies^6–10^. Therefore, further research is important to deepen our understanding of these interactions.

In recent years, the rapid advancement of single-cell RNA sequencing (scRNA-seq) technology has provided unprecedented opportunities to dissect the intricate features of the TME^11–13^. Notably, an increasing number of scRNA-seq-related tools and strategies have been developed to increase the accuracy of bioinformatic analyses and expand analytical dimensions, including batch effect correction, cell-cell interaction assessment, and cellular trajectory inference^14^. To date, the application of single-cell transcriptomics has revealed the biological characteristics of TAMs across various cancer types, such as glioblastoma^15,16^, gastric cancer^7^, breast cancer^17^, colorectal cancer^18^, hepatocellular carcinoma^19^, and clear cell renal cell carcinoma^20^. Furthermore, three recent unbiased studies leveraging single-cell transcriptomic data have systematically investigated the intrinsic features and functions of TAMs at the pan-cancer level, revealing the influence of tumor and environmental factors on macrophage phenotypes and their implications for immunotherapy^21–23^. However, a significant limitation of scRNA-seq is the loss of spatial information during tissue dissociation, which hinders further exploration of the crosstalk between TAMs and other TME components. Fortunately, the recent development of spatial transcriptomics (ST) has addressed this limitation by enabling the acquisition of whole-transcriptome data within tissue sections while preserving spatial context^24^. Consequently, the integration of scRNA-seq and ST data holds great promise for delineating the spatial distribution patterns of TAMs^25^ and facilitating a deeper understanding of cell-cell communication between TAMs and other TME components. Moreover, with the growing popularity of scRNA-seq and ST, an increasing volume of sequencing data has become publicly available, enabling large-scale integration and further in-depth investigations.

By integrating publicly available single-cell and spatial transcriptomic datasets from 16 human cancer types, we constructed a pan-cancer atlas of tumor-associated macrophages across tumor, adjacent normal, and healthy tissues. This analysis covered more than one million cells and 79 spatial transcriptomic sections, enabling the systematic identification of TAM subtypes, their functional programs, clinical relevance, and spatial organization within the TME. Importantly, beyond subtype characterization, we focused on delineating conserved principles of TAM-centered crosstalk with other TME components. Our results demonstrate that bidirectional TAM-TME communication is a pervasive and pan-cancer feature closely linked to tumor progression, providing an integrated framework for understanding TAM biology and informing the development of TAM-targeted therapeutic strategies.

## Results

### Construction of a pan-cancer transcriptomic landscape of the TME

**Fig. 1:**
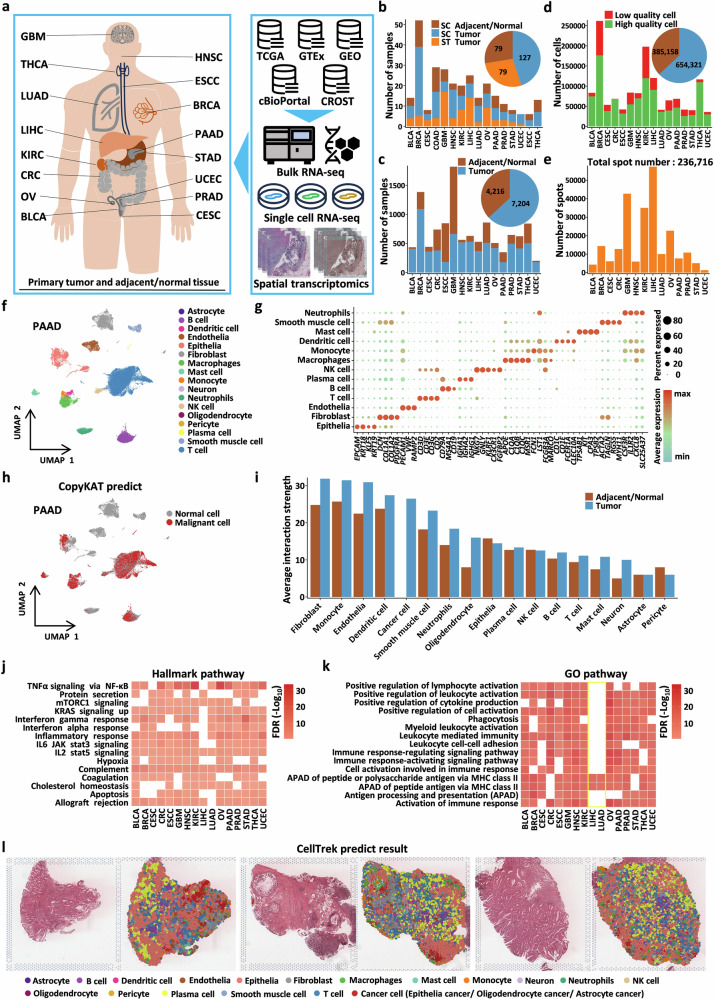
Construction of a pan-cancer transcriptomic landscape of the tumor microenvironment. **a** Data types and sources in this study. The left side lists the cancer types included in this pan-cancer study, while the right side shows all the data types and source platforms used. Some graphical elements were sourced from the iconfont platform. **b** Sample numbers and tissue types for scRNA-seq and ST. Blue represents scRNA-seq tumor samples, brown represents scRNA-seq adjacent normal or healthy tissue samples, and orange represents ST samples. **c** Sample numbers and tissue types for bulk RNA-seq. Blue represents bulk RNA-seq tumor samples, and brown represents bulk RNA-seq adjacent normal or healthy tissue samples. **d** Quality control results for scRNA-seq. Red represents filtered low-quality cells, green represents retained cells, blue represents the number of cells retained in tumor samples, and brown represents the number of cells retained in adjacent normal or healthy tissue samples. **e** Number of spots identified in ST samples. **f** UMAP plot showing major cell types in a single cancer type example, with different colors representing different cell types. **g** Bubble heatmap displaying the expression of marker genes for major cell types. **h** UMAP plot showing malignant cells inferred using copykat. Red represents cells with malignant features, and gray represents cells without malignant tendencies. **i** Bar plot showing the interaction strengths between macrophages and different tumor microenvironment components in normal or adjacent tissues versus tumor tissues inferred using CellChat. **j**, **k** Functional enrichment analysis of macrophages in different cancer types, with pathways sourced from Gene Ontology (GO) and Hallmark. **l** Spatial transcriptomic sample with single-cell resolution inferred and visualized using CellTrek.

To establish a spatially resolved single-cell transcriptomic landscape, we integrated scRNA-seq data from 16 cancer types and ST data from 14 cancer types from the Gene Expression Omnibus (GEO) and Comprehensive Repository of Spatial Transcriptomics (CROST) databases (Supplementary Tables S1 and S2). Additionally, we incorporated bulk RNA-seq datasets and corresponding clinical information for the same cancer types from The Cancer Genome Atlas (TCGA), Genotype-Tissue Expression (GTEx), and cBio Cancer Genomics Portal (cBioPortal) for subsequent validation (Fig. 1a; Supplementary Tables S3, S4, and S5). All the samples were derived from primary tumor tissues and matched adjacent normal or healthy tissues. The scRNA-seq dataset comprised 212 samples, including 127 tumor tissue samples and 79 adjacent normal or healthy tissue samples. The ST dataset included 79 tumor tissue sections (Fig. 1b). The bulk RNA-seq dataset consisted of 11,420 samples, including 7204 tumor tissue samples and 4216 adjacent normal or healthy tissue samples (Fig. 1c). To minimize batch effects caused by differences in sequencing platforms and methodologies, all single-cell and spatial transcriptomic data were obtained exclusively from the 10x Genomics and 10x Visium platforms. Following stringent quality control and filtering, the scRNA-seq data retained a total of 1,039,479 cells, and the ST data retained 236,716 spots for downstream analysis (Fig. 1d, e).

To minimize batch effects across different cancer datasets, we first independently analyzed the scRNA-seq data for each cancer type before integration. Initially, we applied Harmony to reduce potential batch effects between samples^26^. The results demonstrated that batch effects were successfully mitigated (Local Inverse Simpson’s Index (LISI) > 1) (Supplementary Fig. S1a, b). We subsequently employed graph-based clustering and annotated 17 distinct cell types across the 16 cancer datasets using canonical markers for each cell type (Fig. 1f, g and Supplementary Fig. S1c). While most immune, stromal and epithelial cells were present across all cancer types, we also identified tissue-specific cell types, such as neurons and glial cells, in glioblastoma (GBM) (Supplementary Fig. S2a). Tissue preference analysis for different cell types revealed substantial heterogeneity in cell type preferences across cancers. Importantly, we observed that macrophages exhibited a strong preference for tumor tissues in most cancer types (Supplementary Fig. S2b), highlighting the complexity and critical role of cellular components within the TME. To distinguish malignant cells from non-malignant cells, we used CopyKAT to infer single-cell copy number variation (CNV) landscapes^27^. The application of this algorithm to tumor tissues revealed that most cell clusters with malignant tendencies were enriched within epithelial or glial cell populations, which we defined as cancer cells (Fig. 1h and Supplementary Fig. S3a).

To preliminarily characterize the features of macrophages within the TME, we used CellChat to infer their interaction strength with other cell types^28^. The results revealed that compared with those in adjacent normal or healthy tissues, macrophages in tumor tissues interact more strongly with various TME components. Notably, the strongest interactions were detected between fibroblasts and macrophages, and substantial interactions were also observed between macrophages and endothelial cells as well as tumor cells (Fig. 1i; Supplementary Fig. S3b, c). Furthermore, we performed enrichment analyses to identify pathways that are specifically dysregulated in macrophages within tumor tissues. The results demonstrated that macrophages were broadly enriched in pathways related to complement activation, interferon and inflammatory pathways, as well as immune-related processes such as leukocyte activation, cytokine production, phagocytosis, and antigen processing and presentation, which is consistent with previous literature^29^. Additionally, we identified enrichment in pathways such as mTORC1 signaling, KRAS signaling, TNFα signaling via NF-κB, and the hypoxia response (Fig. 1j, k and Supplementary Fig. S3d). Together, these results suggest that TAMs undergo coordinated transcriptional reprogramming toward metabolically active and immunologically engaged states within the tumor microenvironment. Surprisingly, the enrichment analysis results for liver hepatocellular carcinoma (LIHC) and lung adenocarcinoma (LUAD) were distinctly different from those for other cancers. Further analysis revealed that these two cancer types were significantly enriched in energy-related pathways, such as oxidative phosphorylation, ATP synthesis, and metabolism (Supplementary Fig. S3e, f). This divergence is likely attributable to the unique proportions of macrophage subtypes within these cancers^30^.

Additionally, we employed CellTrek, a computational toolkit that maps single cells from scRNA-seq data to spatial coordinates within tissue sections based on ST data^31^. Unlike ST deconvolution methods, CellTrek enables single-cell resolution at the spatial level by transferring individual cells to their spatial coordinates. We applied this approach to scRNA-seq and ST data from the same cancer types to reconstruct spatial single-cell maps, with colorectal cancer (CRC) data serving as an illustrative example (Fig. 1l).

### Construction and characterization of a large-scale, pan-cancer single-cell transcriptome atlas of TAMs in the TME

**Fig. 2:**
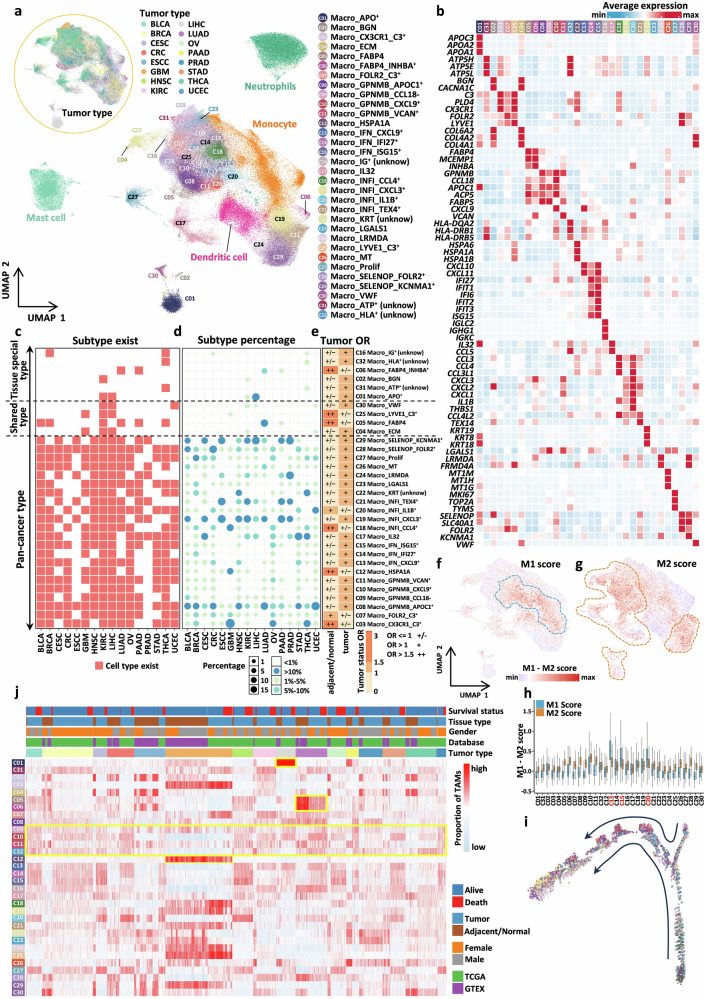
Construction of a large-scale, pan-cancer single-cell transcriptome atlas of TAMs and characterization of TAMs. **a** UMAP plot showing the integration results of all myeloid cells across different cancer types. The top left corner represents the mixing of different cancer types, where C01-C32 denote the 32 defined subtypes. **b** Heatmap displaying the expression of signature genes for different macrophage subtypes. **c** Heatmap depicting the TAM subtypes present across different cancer types. **d** Bubble plot illustrating the relative proportions of different TAM subtypes across cancer types, with both the bubble size and color encoding the magnitude of the proportion. **e** Heatmap showing the preference of different TAM subtypes in tumor tissues, where values greater than 1 indicate significant tissue preference. **f**, **g** UMAP plots displaying the distribution of M1 and M2 scores in TAMs inferred using the AddModuleScore algorithm. **h** Boxplot showing the M1 and M2 scores for different TAM subtypes. Blue represents M1 scores, and orange represents M2 scores. **i** UMAP plot showing the developmental trajectory of macrophages at the single-cell level inferred using Monocle. **j** Heatmap showing the proportions of different TAM subtypes in bulk RNA-seq samples inferred using GSVA. The colored blocks above the heatmap represent different sample information (Supplementary Table S8).

To construct a comprehensive pan-cancer atlas of TAMs that diverges from the conventional M1/M2 classification paradigm, we extracted all macrophage populations from pre-processed single-cell samples across 16 different cancer types. For effective comparative analysis, other myeloid cell types were also isolated and integrated. Subsequent graph-based clustering was performed, revealing no significant bias toward specific tissues or cancer types, with major cell populations clustering cohesively (Fig. 2a; Supplementary Fig. S4a). Subtype nomenclature was assigned based on uniquely highly expressed genes or expression signatures within each cluster (Supplementary Table S6), culminating in the identification of 28 distinct TAM subtypes (Fig. 2a, b). We systematically evaluated the effectiveness of cross-dataset integration using the Single-cell Integration Benchmarking (scIB) framework and assessed the purity of the final subtype definitions with Ratio of Global Unshifted Entropy (ROGUE) scores (Supplementary Fig. S4b, c)^32,33^. Subtypes present in 8 or more cancer types were classified as pan-cancer subtypes, those restricted to 2 or fewer cancer types were classified as tissue-specific subtypes, and the remainder were classified as shared subtypes. Our analysis revealed a predominance of pan-cancer subtypes (21 in total), along with 4 shared subtypes and 3 tissue-specific subtypes (Fig. 2c, d and Supplementary Fig. S4d). Furthermore, ssGSEA enrichment analysis was employed to elucidate the associations between these subtypes and hallmark pathways (Supplementary Fig. S4e).

We first analyzed the low-abundance shared and tissue-specific macrophage subtypes, which are restricted to a limited number of cancer types and thus exhibit pronounced tissue or tumor specificity. Among these subtypes, Macro_APO⁺ (C01), Macro_BGN (C02), Macro_ECM (C04), and Macro_VWF (C30) were clearly enriched in tumor tissues. In contrast, Macro_FABP4 (C05), Macro_FABP4_INHBA⁺ (C06), and Macro_LYVE1_C3⁺ (C25) were significantly enriched in normal and adjacent non-tumor tissues (Fig. 2e).

C02, C04, and C30 all showed pronounced expression of genes associated with extracellular matrix remodeling (e.g., *COL6A2*, *COL4A2*, and *COL4A1*). Their transcriptional profiles closely resembled those of fibroblasts and were predominantly enriched in hepatocellular carcinoma or endometrial carcinoma. Previous studies have suggested that these subtypes may represent transitional populations in which macrophages undergo transdifferentiation toward a fibroblast-like state^34^. In certain tumor microenvironments, this differentiation process may be aberrantly activated, thereby exerting a detrimental effect on the response to immunotherapy. In contrast, C01 represents a liver cancer-enriched, highly specific macrophage subtype characterized by strong expression of multiple lipid metabolism-related genes, including *APOB*, *APOC3*, and *APOA2*, and accounts for a substantial proportion of macrophages in hepatocellular carcinoma. Consistent with the results of our earlier enrichment analyses, the functional characteristics of macrophages in liver cancer are distinct from those observed in other cancer types (Fig. 1k), a phenomenon that may be partly driven by the prominent presence of the C01 subtype.

C05, C06, and C25 exhibited hallmark features of tissue-resident macrophages, including high expression of signature genes such as *C3* and *LYVE1*^30^. Previous studies have shown that C05 and C06 share strong transcriptional similarity with alveolar macrophages in lung tissue^34–37^, whereas C25 more closely resembles a brain-specific macrophage subtype. Although these subtypes are relatively low in abundance within tumor tissues, multiple studies have demonstrated that tissue-resident macrophages of this type are closely associated with tumor invasiveness and responses to immunotherapy^38–41^.

Next, we performed a systematic characterization of the pan-cancer macrophage subtypes, which together accounted for more than two-thirds of the total macrophage population. These subtypes could be robustly identified in more than half of the cancer types analyzed, suggesting that they may share common functional properties across tissues and tumor types. Within this group, five subtypes were predominantly enriched in normal or adjacent non-tumor tissues, whereas seventeen subtypes were significantly enriched in tumor tissues (Fig. 2e). Notably, four representative subtypes emerged: tissue-resident macrophage subsets, inflammation-associated macrophage subsets, chemokine-enriched macrophage subtypes, and a mixed macrophage subtype family characterized by high *GPNMB* expression.

The tissue-resident macrophage subgroup includes Macro_CX3CR1_C3⁺ (C03), Macro_FOLR2_C3⁺ (C07), Macro_HSPA1A (C12), and Macro_SELENOP_FOLR2⁺ (C28). Among these, the *FOLR2*-high subtypes C07 and C28 have been reported to colocalize with CD8⁺ T cells across multiple cancer types, suggesting a potential role in CD8⁺ T-cell activation and anti-tumor immunity^38,42,43^.

The inflammation-associated macrophage subgroup comprises Macro_INFI_TEX14⁺ (C21), Macro_INFI_IL1B⁺ (C20), Macro_INFI_CXCL3⁺ (C19), and Macro_INFI_CCL4⁺ (C18). C20 is characterized by marked upregulation of the expression of *IL1B* and multiple chemokines (e.g., *CXCL3* and *CXCL2*), with *IL1B* recognized as a key pro-inflammatory cytokine secreted by M1-like macrophages; in addition, this subtype is characterized by high expression of the epidermal growth factors *AREG* and *EREG*, suggesting potential involvement in angiogenesis and tumor metastasis^44,45^. C21 specifically overexpresses *TEX14*, a gene closely associated with reproductive development^46^. C19 is characterized by high expression of *MIR155HG* and *PDE4DIP*, of which *MIR155HG* is a peptide-encoding lncRNA involved in inflammation and antigen presentation^47^, whereas *PDE4DIP* has been shown to participate in NF1/RAS signaling and has been proposed as a potential therapeutic target in colorectal cancer^48^. C18 prominently expresses multiple cytokines, including *CCL4*, *CCL4L2*, and *CCL3L3*, which is accompanied by high expression of the interferon-stimulated gene *CH25H*.

The chemokine-enriched macrophage subtypes included Macro_IFN_CXCL9⁺ (C13), Macro_IFN_IFI27⁺ (C14), and Macro_IFN_ISG15⁺ (C15). Among them, C13 is characterized by high expression of the chemokines *CXCL9*, *CXCL10*, and *CXCL11*, particularly *CXCL9*, a key factor known to be closely involved in T-cell recruitment^49,50^. C14 shows marked upregulation of multiple interferon-related genes, including *IFI6*, *IFI27*, and *IFITM3*. Similarly, C15 exhibits concurrent high expression of signature genes from both the chemokine and interferon families. Collectively, these molecular features indicate that these subtypes share substantial functional similarity with classically defined M1-like macrophages^51–53^ and suggest a close functional association with T cells.

Macrophage subtypes characterized by high *GPNMB* expression, including Macro_GPNMB_APOC1⁺ (C08), Macro_GPNMB_CCL18⁻ (C09), Macro_GPNMB_CXCL9⁺ (C10), and Macro_GPNMB_VCAN⁺ (C11), display complex and heterogeneous functional features. Previous studies have shown that *GPNMB* is markedly upregulated during macrophage–tumor interactions and can promote cancer stem cell formation and tumor cell metastasis through *CD44* and *IL-33* signaling; thus, it is emerging as an important potential therapeutic target^54,55^. Among these subtypes, C10 has transcriptional characteristics similar to those of chemokine- and interferon-related macrophage subsets. C08, C09, and C11 are characterized by high expression of the secreted phosphoprotein *SPP1*, which is widely recognized as a hallmark gene of multiple macrophage subtypes and a key pro-tumorigenic factor^7,19,56–58^. In addition, C11 shows pronounced upregulation of genes associated with hypoxia and metabolism, including *BNIP3*, *HK2*, *NUPR1*, *ERO1A*, *ALDOA*, and *ADM*, and is linked to pro-angiogenic processes, suggesting that C11 plays a role in providing metabolic and survival support for tumor growth. In contrast, C08 and C09 also highly express *APOC1*, a lipid metabolism-related gene^59^ that is closely associated with a poor prognosis in multiple cancers^60^ and has been implicated in resistance to immunotherapy^61^.

In addition to the four major pan-cancer macrophage subtypes described above, we identified several macrophage subpopulations with relatively distinct molecular features. Among them, Macro_LRMDA (C24) is characterized by high expression of the melanocyte differentiation-associated gene *LRMDA*. Macro_SELENOP_KCNMA1⁺ (C29) exhibits gene expression patterns suggestive of potential associations with neural-related functions. Macro_Prolif (C27) represents a proliferative subpopulation marked by strong cell cycle-related signals, a feature observed across multiple cell types. Macro_MT (C26) is characterized by high expression of multiple metallothionein genes. In addition, two relatively rare subtypes, Macro_LGALS1 (C23) and Macro_IL32 (C17), were also identified. Notably, all of these subpopulations were consistently enriched in tumor tissues, suggesting that they may play specific and functionally important roles within the tumor microenvironment.

Finally, we excluded four subpopulations likely representing doublets or contamination artifacts. Macro_IG⁺ (C16) strongly expressed B-cell-associated immunoglobulin genes (e.g., *IGKC*), whereas Macro_KRT (C22) expressed epithelial markers (*KRT19*, *KRT18*, and *KRT8*), which is consistent with the doublet signatures. In contrast, Macro_ATP⁺ (C31) and Macro_HLA⁺ (C32) were dominated by nonspecific ATP metabolism- and HLA-related signals, respectively, which is indicative of background contamination.

After thoroughly characterizing the signature genes and potential biological functions associated with different TAM subtypes, we sought to infer the M1/M2 binary polarization states of TAMs. To this end, we referenced previously reported M1 and M2 signature genes from the literature (Supplementary Table S7)^22^ and employed the AddModuleScore algorithm to calculate M1 and M2 scores for each subtype (Fig. 2f-h). Although the polarization tendencies varied across subtypes (with C13-Macro_IFN_CXCL9^+^, C15-Macro_IFN_ISG15^+^, and C20-Macro_INFI_IL1B^+^ exhibiting pronounced M1 characteristics), it is undeniable that the majority of subtypes displayed co-expression of both M1 and M2 signature genes. This observation aligns with findings reported in previous studies^62^.

The complex characteristics of TAMs within the TME can be attributed to their intricate developmental processes. To infer the developmental trajectory of TAMs, we utilized the Monocle2 algorithm^63^. To minimize the influence of subtypes with pronounced tissue-specific biases, we selected single-cell data from the 21 pan-cancer subtypes to construct the developmental trajectory. We observed that the overall trajectory originates from two starting points and ultimately converges toward a common direction (Fig. 2i and Supplementary Fig. S4f). TAMs with tissue-resident macrophage (TRM) characteristics were positioned at the initial stage of development, while the majority of the subtypes were located in the intermediate stage. Notably, two specific inflammatory subtypes (Macro_INFI_CXCL3^+^ and Macro_INFI_TEX4^+^), as well as Macro_LRMDA and Macro_SELENOP_KCNMA1^+^, were present at the terminal stage of development (Supplementary Fig. S4g). Further analysis revealed that the developmental axis involves the regulation and activation of complex biological processes, including wound healing, granulocyte chemotaxis, T-cell activation, response to oxidative stress, Ras protein signal transduction, inflammatory response, and response to TGF-β (Supplementary Fig. S4h, i). This highlights the inherent heterogeneity of TAMs and their critical role in activating diverse biological functions within the organism.

Finally, to establish a connection between the scRNA-seq and traditional bulk RNA-seq samples, we employed the ssGSEA algorithm to perform deconvolution based on the signature gene sets of the 28 subtypes, thereby inferring the abundance of TAMs in the bulk RNA-seq samples (Fig. 2j and Supplementary Table S8). Our results revealed features consistent with those observed at the single-cell level, such as the enrichment of Macro_APO^+^ (C01) in liver tissues and Macro_FABP4 (C05 and C06) in lung tissues, further validating the reliability of our previous findings.

### TAM co-occurrence patterns and correlation with clinical features

**Fig. 3:**
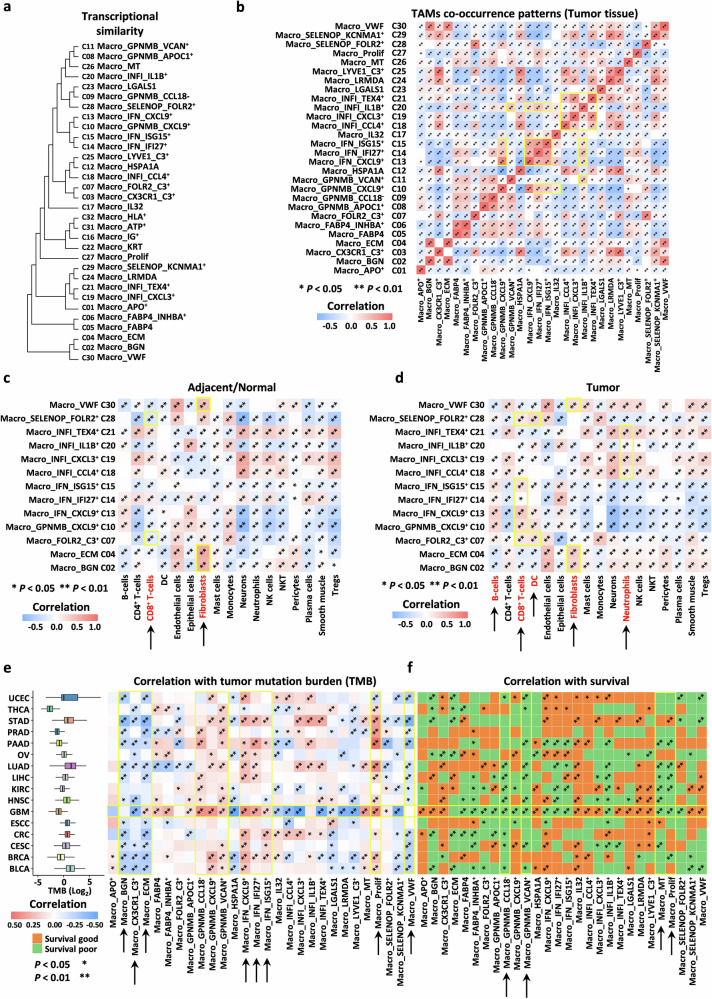
TAM co-occurrence patterns and correlation with clinical features. **a** Dendrogram showing the similarity clustering of different TAM subtypes. **b** Pearson correlation analysis of the co-occurrence patterns of different TAM subtypes in tumor tissues (*P < 0.05, **P < 0.01). **c**, **d** Pearson correlation analysis of the co-occurrence patterns between different hub TAM subtypes and TME components (inferred by xCell) in normal or adjacent tissues (**c**) and tumor tissues (**d**) (*P < 0.05, **P < 0.01). **e** Left: boxplot showing the average tumor mutation burden (TMB) across different cancer types; Right: heatmap representing the correlation between different TAM subtypes and TMB (*P < 0.05, **P < 0.01). **f** Heatmap representing the correlation between different TAM subtypes and prognosis (*P < 0.05, **P < 0.01).

Given that we constructed a comprehensive pan-cancer TAM atlas, we sought to explore the phenotypic relationships among these subtypes. Using unsupervised hierarchical clustering analysis, we identified 10 major branches (Fig. 3a). Contrary to our expectations, some subtypes expressing similar signature genes did not cluster together, highlighting their transcriptional heterogeneity. For instance, two subtypes with high expression of *GPNMB* (C08 and C11) formed a distinct branch alongside C26 and the inflammatory subtype C20. Several subtypes characterized by high expression of chemokine- and interferon-related signatures clustered into a unified branch (C10, C13, C14, and C15). Subtypes with tissue-resident features or those enriched in normal tissues formed a separate branch (C25, C12, C10, C07, and C03). Additionally, the three subtypes associated with tissue stroma (C02, C04, and C30) and the two subtypes recruited to lung tissue (C05 and C06) each formed distinct branches.

We further explored the co-occurrence patterns among TAMs using Pearson correlation analysis of cluster frequencies to infer potential intrinsic relationships between different subtypes (Supplementary Fig. S5a). For instance, several subtypes with high expression of chemokine- and interferon-related signature genes exhibited stronger positive correlations in tumor tissues, suggesting their co-occurrence within the TME. Similarly, subtypes characterized by inflammatory signature genes also displayed comparable patterns (Fig. 3b). These co-occurrence results demonstrate the existence of coordinated interactions among different TAM subtypes. Additionally, we analyzed the co-occurrence relationships between TAMs and other components of the TME. Since we were unable to comprehensively define all TME components at the single-cell level, we employed the xCell algorithm^64^ to infer the abundance of different cell types in the samples (Supplementary Fig. S5b and Table S9). We subsequently constructed a co-occurrence network using Pearson correlation analysis of cluster frequencies (Supplementary Fig. S5c). We found that macrophages exhibit complex co-occurrence patterns with other TME components. For example, the three subtypes associated with tissue stroma (C02, C04, and C30) consistently co-occurred with fibroblasts in both normal and primary tumor tissues. Subtypes with high expression of chemokine-related signatures (C10, C13, C14, and C15) strongly co-occurred with CD8^+^ T cells, suggesting their potential role in T-cell recruitment. In contrast, inflammatory subtypes (C18, C19, C20, and C21) co-occurred with neutrophils (Fig. 3c, d). These findings indicate potential interactions among different TAM subtypes, but the intricate co-occurrence network requires further exploration to elucidate its underlying biological characteristics.

The tumor mutational burden (TMB) is a quantitative biomarker that reflects the total number of mutations carried by tumor cells in the genome. The TMB is closely associated with the activity of immune checkpoint inhibitors (ICIs) and is widely recognized as a biomarker for immunotherapy, with higher TMB values often indicating greater benefit from immunotherapy. We first downloaded quantified TMB data from cBioPortal (Supplementary Table S5) and calculated the correlation between the abundance of different subtypes and TMB. We identified several subtypes with high expression of *GPNMB* and chemokine-related signatures, as well as Macro_Prolif, which showed significant positive correlations with the TMB. In contrast, subtypes such as Macro_BGN, Macro_ECM, Macro_CX3CR1_C3^+^, and Macro_VWF exhibited significant negative correlations (Fig. 3e). Therefore, focusing on subtypes with strong TMB correlations is crucial for identifying effective immunotherapy targets. We subsequently analyzed the associations between the inferred abundance of TAMs in the bulk RNA-seq data and patient prognosis across different cancer types. We found that most subtypes often exhibit varying survival outcomes across different cancers. Notably, Macro_GPNMB_VCAN^+^, Macro_GPNMB_CCL18^-^, Macro_MT, and Macro_Prolif were associated with a poor prognosis in most cancers, and GBM appeared to be particularly sensitive to changes in macrophage abundance, with prognostic correlations with nearly all subtypes (Fig. 3f).

### Pan-cancer spatial distribution characteristics of TAMs

**Fig. 4:**
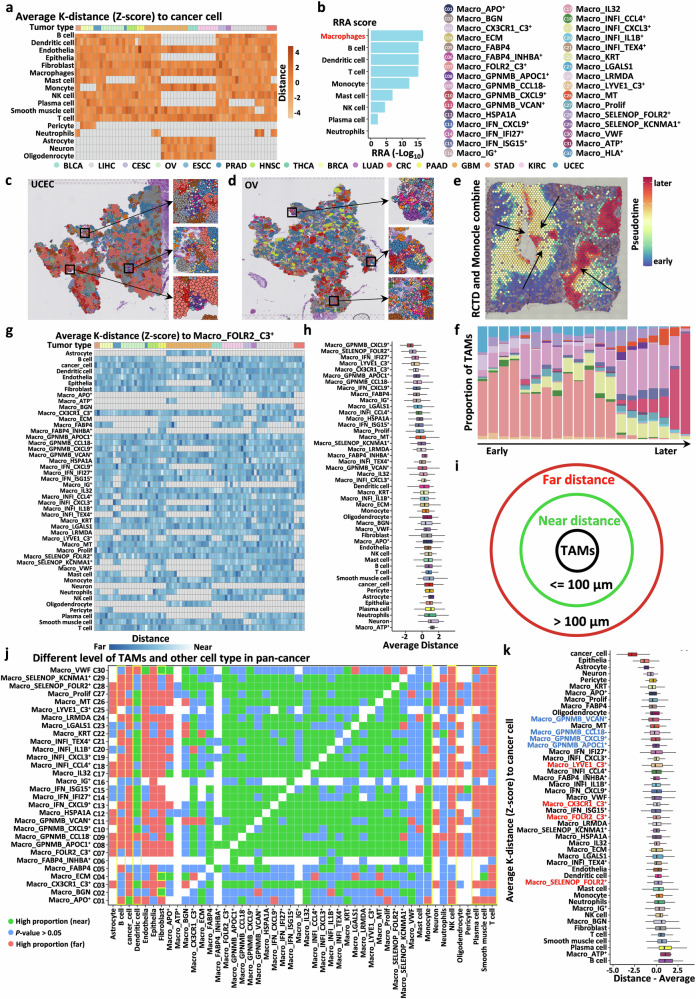
Pan-cancer spatial distribution characteristics of TAMs. **a** Heatmap showing the average distance of different cell types to cancer cells on each tissue slice, with columns transformed using z-score; the colors at the top represent the tumor types. **b** Ranking of immune cells based on their proximity to cancer cells across 79 slices using the RRA algorithm, ordered from closest to farthest. **c**, **d** Distribution maps of cell types on tissue slices embedded with TAM subtypes, visualized using CellTrek. **e** Inference of TAM developmental trajectories at the spatial level using RCTD combined with the Monocle2 algorithm. **f** Bar plot showing the proportions of different cell types along the developmental time axis. The x-axis represents the developmental time from early to late, and the color shading indicates the proportion of different TAM subtypes at each time point. **g** Heatmap showing the average distance of different cell types to Macro_FOLR2_C3^+^ cells on each tissue slice, with columns transformed using z-score. The colors at the top represent the tumor types. **h** Boxplot showing the average distance of different cell types to Macro_FOLR2_C3^+^ cells across 79 slices at the pan-cancer level, ordered from closest to farthest. **i** Circular plot displaying the proportions of cells in the proximal and distal regions relative to the number of TAMs. **j** Heatmap showing the enrichment of various cell types in the proximal and distal regions of each TAM subtype. Paired *t*-tests were used to compare the differences in cell proportions between the proximal and distal regions of each TAM subtype. Red indicates enrichment of cell types in the distal region of TAMs, whereas green indicates enrichment in the proximal region. Blue represents no statistical significance (*P* > 0.05). **k** Boxplot showing the average distance of different cell types to cancer cells across 79 slices at the pan-cancer level, ordered from closest to farthest.

We first calculated the average spatial distances between different cell types based on CellTrek localization results. To mitigate the influence of varying tissue slice sizes, we applied z-score normalization to samples from the same tissue slice. Robust Rank Aggregation (RRA) is an algorithm designed to integrate rankings and generate a consolidated ranking list^65^. We ranked the average distances between different immune cell types and cancer cells and used the RRA algorithm to integrate these rankings, obtaining a comprehensive ranking for all immune cells. The results revealed that macrophages exhibited the closest spatial proximity to cancer cells, further underscoring their critical role in tumor progression (Fig. 4a, b).

Next, to determine the spatial distribution characteristics of the TAM subtypes we defined, we annotated the cell subpopulations and integrated this information into the CellTrek object. We observed significant differences in the spatial distribution patterns among the subtypes (Fig. 4c, d), with high-density regions often localized to distinct spatial areas, suggesting that the activation state of TAMs is influenced by their position within the TME. To further explore this, we employed RCTD^66^ combined with the Monocle2^63^ algorithm to infer the developmental trajectory of TAMs at the spatial level, using one GBM tissue slice as an example. The results demonstrated that cell types and their proportions evolved along the trajectory (Fig. 4e, f), accompanied by the emergence of various biological features, such as hypoxia response (Cluster 8), extracellular matrix remodeling (Cluster 1), and antigen presentation (Cluster 3) (Supplementary Fig. S6a, b). These findings collectively indicate that the functional state and biological activation of TAMs are influenced by their specific spatial context within the TME.

To further quantify spatial relationships, we aimed to define the spatial distances between different subtypes at the pan-cancer level (Fig. 4g). Since most subtypes could not be mapped to all tissue slices, we calculated the average distances across different slices to determine the spatial distances between TAM subtypes and other cell types at the pan-cancer level (Fig. 4h and Supplementary Fig. S7a–o, S8a–o). Notably, most TAM subtypes exhibited relatively small spatial distances from one another. Monocytes and DCs, which share high similarity with macrophages, were also found in close proximity to most subtypes. Additionally, we observed that many immune cells, such as T cells, B cells, and plasma cells, maintained greater spatial distances from TAMs. This spatial separation may contribute to the immunosuppressive microenvironment often associated with TAMs.

To better investigate the microenvironmental characteristics surrounding different TAM subtypes, we defined cells within 100 μm of spatial distance to TAMs on the same tissue slice as “proximal cells”, while the remaining cells were classified as “distal cells” (Fig. 4i). We then calculated the proportions of different cell types among “proximal cells” and “distal cells”. We subsequently used paired *t*-tests to compare the differences in cell type proportions between the proximal and distal regions around macrophages at the pan-cancer level (Fig. 4j). As expected, most TAM subtypes were enriched with other TAM subtypes in their proximal regions. Additionally, we found that most anti-tumor immune cell populations exhibited pronounced spatial exclusion from TAM subtypes. Beyond these general patterns, we also identified several unique spatial relationships. For instance, Macro_ECM, Macro_VWF, and Macro_BGN were enriched with fibroblasts, suggesting that these subtypes may represent intermediate states in the differentiation of macrophages into CAFs. Finally, we identified a group of subtypes surrounded by a high density of cancer cells (Fig. 4k), including Macro_APO^+^, Macro_FABP4, and Macro_GPNMB_VCAN^+^. These findings further suggest that these subtypes may exhibit strong physical interactions with cancer cells.

### TAMs promote CD8^+^ T-cell activation and represent potential key targets for tumor immunotherapy

**Fig. 5:**
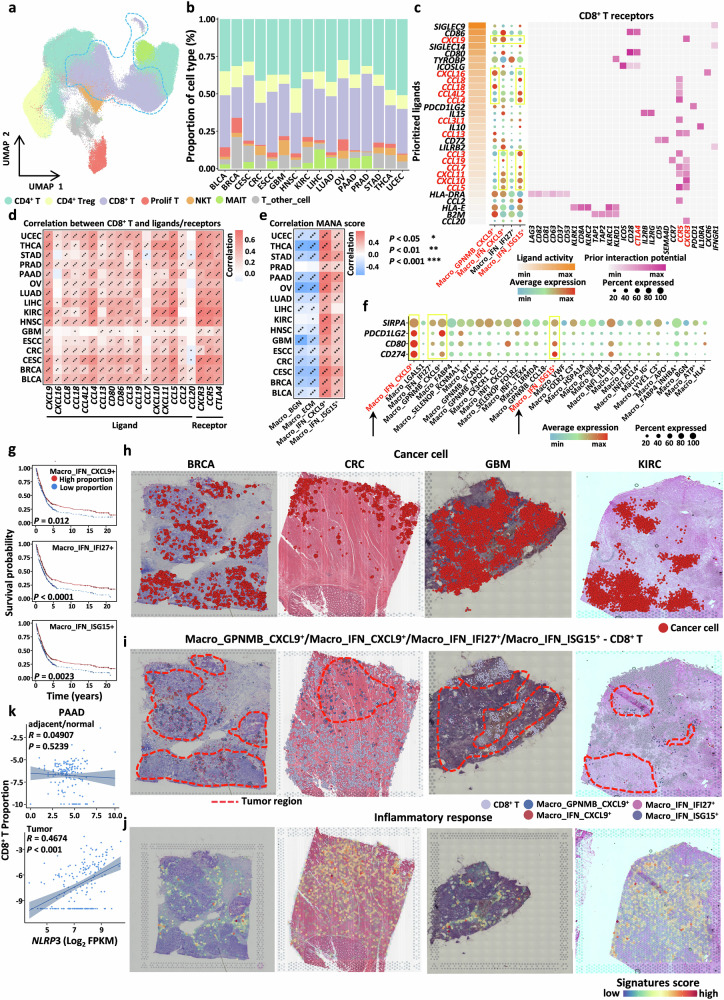
TAMs promote CD8^+^ T-cell activation and represent potential key targets for tumor immunotherapy. **a** UMAP plot showing the integration results of T cells across different cancer types. **b** Bar plot displaying the proportions of different T-cell subtypes in various cancer types. **c** Left: heatmap showing the activity scores of different ligands, sorted in descending order. Middle: bubble plot showing the expression levels of different ligands in IFN^+^ Macro subtypes. Right: Heatmap showing the interaction strength between ligands and receptors. **d** Heatmap displaying the correlation between receptors and ligands involved in TAM-CD8^+^ T-cell interactions and the abundance of CD8^+^ T cells (*P < 0.05, **P < 0.01, ***P < 0.001). **e** Heatmap showing the correlation between different TAM subtypes and mutation-associated neoantigen (MANA) scores (*P < 0.05, **P < 0.01, ***P < 0.001). **f** Bubble plot displaying the expression of immune checkpoint genes in major TAM subtypes. **g** Kaplan‒Meier curves showing the prognostic value of three TAM subtypes in immunotherapy cohorts, with *P* values calculated using the log-rank test. **h** Distribution of cancer cells inferred using CellTrek. **i** Distribution of four subtypes with high expression of chemokines inferred using CellTrek; the red dashed area represents the primary distribution region of cancer cells annotated based on Fig. 5h. **j** Spatial slice showing the scoring map of inflammatory pathways. **k** Correlations between CD8^+^ T cells and *NLRP3* expression in normal or adjacent tissues and tumor tissues.

One of the critical features of macrophages in the TME is their involvement in immune processes (Fig. 1j, k), while CD8^+^ T cells play a pivotal role in anti-tumor immunity^67–69^. However, the mechanisms underlying the interactions between TAMs and CD8^+^ T cells remain elusive. We identified two main subtypes of TAMs that significantly co-occurred with CD8^+^ T cells (Fig. 3d): the FOLR2^+^ Macro subtypes (Macro_FOLR2_C3^+^ and Macro_SELENOP_FOLR2^+^), which are characterized by high expression of *FOLR2*, and the IFN^+^ Macro subtypes (Macro_GPNMB_CXCL9^+^, Macro_IFN_CXCL9^+^, Macro_IFN_IFI27^+^, and Macro_IFN_ISG15^+^), which highly express chemokine-related genes. Notably, these two subtypes are widely present across most tissue types (Fig. 2c, d). To elucidate the interactions between these TAM subtypes and CD8^+^ T cells in detail, we integrated T cells from all the samples and defined the CD8^+^ T-cell subtypes (Fig. 5a and Supplementary Fig. S9a, b). We found that CD8^+^ T-cell subtypes accounted for a significant proportion of all cancer types (Fig. 5b), further underscoring their critical role in tumor immunity.

FOLR2^+^ Macro subtypes have been reported to co-localize with CD8^+^ T cells, effectively activating CD8^+^ T cells, and are associated with favorable patient outcomes in multiple cancers^38,42,43^. Compared with their negative correlation in normal tissues (Fig. 3c), the co-occurrence of the FOLR2^+^ Macro subtypes with CD8^+^ T cells was more significant in tumor tissues (Fig. 3d), suggesting stronger interactions between these subtypes and CD8^+^ T cells within the TME. Functional enrichment analysis further confirmed that the FOLR2^+^ Macro subtypes were involved in antigen presentation and the activation and regulation of T cells (Supplementary Fig. S9c, d). Prognostic analysis revealed that a high abundance of both FOLR2^+^ Macro and CD8^+^ T cells was significantly associated with favorable outcomes in multiple cancers (Supplementary Fig. S9e). Additionally, FOLR2^+^ Macro subtypes co-occurred with B cells and DCs in tumor samples (Fig. 3d), which is consistent with previous reports^38^. Therefore, FOLR2^+^ Macro subtypes may possess strong anti-tumor immune potential.

To identify key regulatory factors mediating the interactions between the IFN^+^ Macro and CD8^+^ T-cell subtypes, we employed the NicheNet software package to investigate the underlying mechanisms of these cellular interactions^70^. We observed that CD8^+^ T cells exhibit high activity in response to a series of chemokines expressed by the IFN^+^ Macro subtype, including *CXCL9, CXCL10*, *CXCL11*, *CXCL16*, *CCL8*, and *CCL4L2*. These ligands bind to the receptors *CCR5*^51,71,72^ and *CXCR3*^73,74^ expressed on CD8^+^ T cells (Fig. 5c). These findings suggest that the IFN^+^ Macro subtype plays a role in recruiting CD8^+^ T cells. To further validate this, we performed correlation analysis using bulk RNA-seq datasets and found strong positive correlations between the expression levels of these ligands and receptors and the abundance of CD8^+^ T cells (Fig. 5d).

The interaction between TAMs and CD8^+^ T cells can influence the response to ICIs in tumor therapy. We aimed to further explore the relationship between TAMs and the tumor immunotherapy response. We integrated immunotherapy data from 1229 cases across 10 cancer types, along with corresponding expression profiles and clinical data (Supplementary Table S10). We observed that for most malignancies, the proportion of immunotherapy responders did not exceed 50%, indicating the limited efficacy of immunotherapy (Supplementary Fig. S9f, g). First, we calculated the abundance of TAM subtypes in immunotherapy-treated samples using the ssGSEA algorithm based on their signature genes. Interestingly, we found that three IFN^+^ Macro subtypes (Macro_IFN_CXCL9^+^, Macro_IFN_IFI27^+^, and Macro_IFN_ISG15^+^) were enriched in immunotherapy responders (Supplementary Fig. S9h). Concurrently, we identified two subtypes (Macro_ECM and Macro_BGN) that were most enriched in non-responders (Supplementary Fig. S9h). The mutation-associated neoantigen (MANA) scores were used to evaluate changes in the transcriptional programs of T cells stimulated by cancer-associated neoantigens (Supplementary Table S11)^75^. We selected the four TAM subtypes most enriched in responders and non-responders and performed correlation analysis (*P*-value < 0.001), revealing that Macro_IFN_CXCL9^+^ and Macro_IFN_ISG15^+^, which were enriched in responders, were positively correlated with MANA scores, whereas Macro_ECM and Macro_BGN were negatively correlated with MANA scores (Fig. 5e). These analyses suggest that IFN^+^ Macro subtypes may increase the sensitivity of the TME to ICI therapy. The results of previous correlation analyses with the TMB revealed similar conclusions (Fig. 3e). To assess the potential clinical relevance of these findings, we investigated whether these subtypes express known immune checkpoints. We found that several key immune checkpoints (e.g., *CD274*, *CD80*, and *PDCD1LG2*)^76^ were most highly expressed in Macro_IFN_CXCL9^+^ and Macro_IFN_ISG15^+^ cells, followed by Macro_IFN_IFI27^+^ and Macro_GPNMB_CXCL9^+^ cells (Fig. 5f). Additionally, survival analysis indicated that a high abundance of three of these subtypes was associated with a favorable prognosis in immunotherapy-treated patients (Fig. 5g).

However, unexpectedly, in some ST data with high IFN^+^ Macro abundance, we observed that CD8^+^ T cells were recruited to the periphery of cancer cells rather than infiltrating the tumor core, suggesting that the tumor-killing capacity of CD8^+^ T cells was limited in these samples (Fig. 5h, i). It has been demonstrated in liver cancer that TAMs expressing high levels of *CXCL9* and *CXCL10* can recruit and retain CD8^+^ T-cell-derived reactive cytotoxic T lymphocytes (CTLs), thereby promoting the formation of an immunosuppressive environment within tumors^77^. Further analysis revealed elevated inflammatory levels around CD8^+^ T cells and these chemokine-high subtypes (Fig. 5j). Through a pan-cancer correlation analysis (Fig. 5k and Supplementary Fig. S10a, b), we found that compared with that in adjacent normal tissues, the infiltration level of CD8^+^ T cells in tumor tissues was positively correlated with the expression of the inflammasome (*NLRP3*), the activation of which has been shown to promote further cancer progression^78^.

### TAMs localized in the tumor core participate in angiogenesis and metabolic reprogramming

**Fig. 6:**
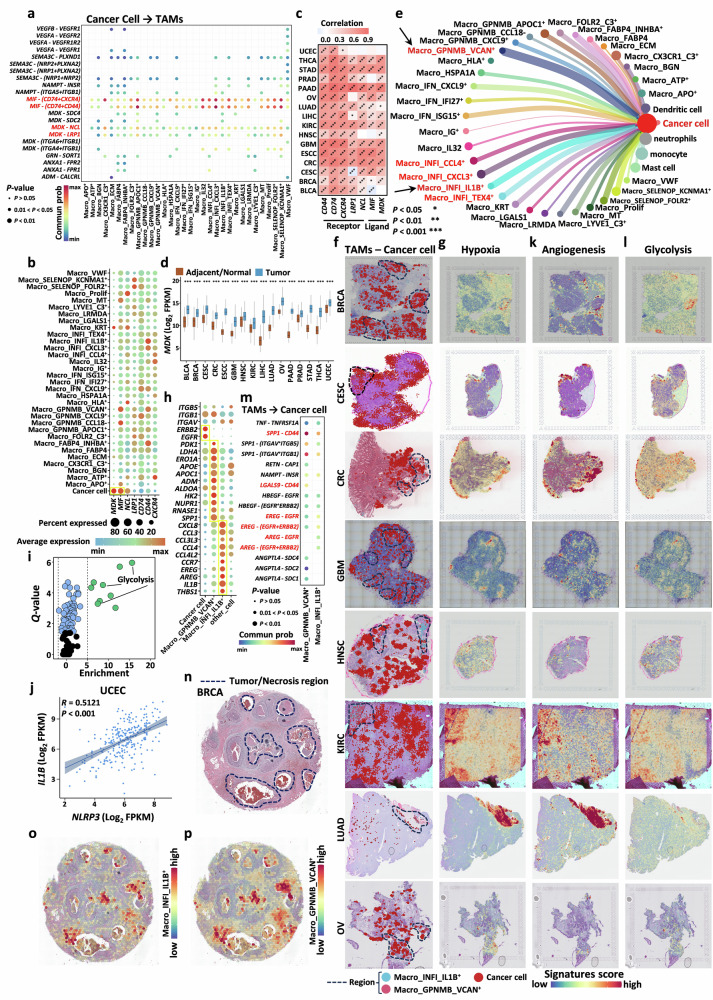
TAMs localized in the tumor core participate in angiogenesis and metabolic reprogramming. **a** Bubble plot showing the interactions between cancer cells and TAMs, along with ligand-receptor pairs. **b** Bubble plot showing the expression of *MDK*, *MIF*, and their corresponding receptors in TAMs. **c** Heatmap displaying the correlation analysis between the key ligands *MIF* and *MDK*, their receptors, and the M2 scores of the samples (*P < 0.05, **P < 0.01, ***P < 0.001). **d** Boxplot showing the differential expression of *MDK* at the pan-cancer level (*P < 0.05, **P < 0.01, ***P < 0.001). **e** Dot plot showing the interaction strength between different TAM subtypes and cancer cells, with the thickness of the connecting lines representing the strength of the interactions. **f** CellTrek visualization showing the spatial distribution of Macro_GPNMB_VCAN^+^, Macro_INFI_IL1B^+^, and cancer cells in tissue slices; the purple dashed area represents the primary distribution region of Macro_GPNMB_VCAN^+^ and Macro_INFI_IL1B^+^ cells. **g** Spatial distribution maps of hypoxia pathways in tissue slices. **h** Bubble plot displaying the expression of signature genes in Macro_GPNMB_VCAN^+^, Macro_INFI_IL1B^+^ and cancer cells. **i** SCPA analysis of metabolic pathways in Macro_GPNMB_VCAN^+^ cells. **j** Correlations between *IL1B* and *NLRP3* expression. **k**, **l** Spatial distribution maps of the angiogenesis- and glycolysis-related pathways in tissue slices. **m** Bubble plot showing the interactions between TAMs and cancer cells, along with ligand-receptor pairs. **n** Annotation of tumor necrosis regions in breast cancer slices from an external dataset; the purple dashed area represents the tumor necrosis region. **o**, **p** Spatial scores of Macro_GPNMB_VCAN^+^ and Macro_INFI_IL1B^+^ cells in an external dataset.

We focused on potential interactions between TAMs and malignant cancer cells. Using CellChat software, we inferred the interaction axes between them and identified two universally present axes: *MIF*-(*CD74* + *CXCR4*/*CD44*) and *MDK*-(*NCL*/*LRP1*) (Fig. 6a). *MDK* has been reported to activate immunosuppressive macrophages in gallbladder cancer^79^, whereas *MIF* is widely involved in the regulation of macrophage functions^80,81^. Our data revealed that both *MDK* and *MIF* were highly expressed specifically in cancer cells, whereas their corresponding receptors were broadly expressed across most TAM subtypes (Fig. 6b). *MDK* and *MIF* have been implicated in M2 macrophage polarization in metastatic models of non-small cell lung cancer^82^ and gastric cancer models^83^, respectively. We sought to further investigate their polarization potential at the pan-cancer level. Using M2 signature genes collected from the literature, we inferred M2 scores for bulk RNA-seq samples using the ssGSEA algorithm (Supplementary Table S7) and calculated the correlations between *MDK*, *MIF*, their corresponding receptors, and M2 scores (Fig. 6c and Supplementary Fig. S11a). The analysis revealed strong positive correlations between these factors and M2 polarization in most cancers. Additionally, *MDK* and *MIF* were significantly overexpressed in tumor samples at the pan-cancer level (Fig. 6d and Supplementary Fig. S11b), suggesting that targeting these genes to inhibit the M2 polarization of TAMs could be a feasible strategy for tumor therapy.

The functional relationship between TAMs and cancer cells was also a focus of our study. We found that Macro_GPNMB_VCAN^+^ cells exhibited the most significant interactions with cancer cells (Fig. 6e), followed by inflammatory subtypes, particularly Macro_INFI_IL1B^+^ cells, which displayed the most representative features (Supplementary Fig. S4e). These two subtypes also showed spatial proximity (Supplementary Figs. S7k and S8e) and were associated with hypoxia-related features (Supplementary Fig. S4e). ST data confirmed their localization in hypoxic regions of tumors (Fig. 6f, g). The core regions of tumors are often characterized by hypoxia and angiogenesis^84–86^, suggesting that these subtypes may directly contribute to tumor progression. Additionally, the hypoxic microenvironment functionally suppresses the cell cycle and oxidative phosphorylation and is associated with the inhibition of T-cell immune activity and drug resistance^15^. Macro_GPNMB_VCAN^+^ cells were surrounded by a high density of cancer cells (Fig. 4k). In addition to hypoxia-related features, the signature genes of this subtype (e.g., *PDK1*, *LDHA*, *ERO1A*, *ADM*, *ALDOA*, and *HK2*) are associated with glycolysis and lactate production pathways (Fig. 6h; Supplementary Fig. S11c). SCPA analysis^87^ confirmed that glycolysis was the most prominent metabolic pathway, indicating that TAMs in the tumor core may support cancer cells by promoting metabolic reprogramming to meet their energy demands (Fig. 6i). In contrast, Macro_INFI_IL1B^+^ cells, in addition to the inflammatory signature genes, highly express angiogenesis-related genes such as *THBS1*, *AREG*, and *EREG* (Fig. 6h). Enrichment analysis further revealed that Macro_INFI_IL1B^+^ cells can recruit neutrophils (Supplementary Fig. S11d). Since *IL1B* is a known key factor in neutrophil recruitment, cell communication analysis confirmed that it recruits and activates neutrophils through the *IL1B*-*IL1R2* and *CXCL8*-*CXCR2* interaction axes^88–91^ (Supplementary Fig. S11e, f). Moreover, correlation analysis indicated that *IL1B* has the potential to activate *NLRP3* (Fig. 6j; Supplementary Fig. S11g). Thus, Macro_INFI_IL1B^+^ cells may also promote tumor angiogenesis by recruiting neutrophils. At the spatial level, regions enriched with these two subtypes exhibited high concentrations of features related to angiogenesis and glycolysis (Fig. 6k, l). Cell communication analysis revealed that Macro_GPNMB_VCAN^+^ cells primarily interact via the *SPP1/LGALS9*-*CD44* axis^92–95^, whereas Macro_INFI_IL1B^+^ cells interact through the *EREG*-(*EGFR* + *ERBB2*) and *AREG*-(*EGFR* + *ERBB2*) axes, which are closely associated with tumor invasion and metastasis (Fig. 6m). Finally, we also observed necrotic features in the regions enriched with these two subtypes (Supplementary Fig. S11h), which was further validated in an external dataset (Fig. 6n–p), suggesting their association with rapid tumor growth.

The results of the above analyses highlight the close relationship between TAMs and tumor metabolism. A growing body of research has reported the critical role of metabolic reprogramming in TAM-mediated tumor progression, particularly in inflammatory or hypoxic environments^96–101^. To extend metabolic characteristics to the entire cell population and infer general patterns linking TAMs to metabolic reprogramming, we performed scMetabolism analysis^102^ and identified various metabolic reprogramming mechanisms associated with tumor growth across different TAM subtypes. TAMs enriched in the tumor periphery or adjacent regions, including Macro_APO^+^, Macro_FABP4, Macro_Prolif, Macro_GPNMB_VCAN^+^, Macro_GPNMB_CCL8^-^, and Macro_GPNMB_APOC1^+^ cells, appear to exhibit increased activity in metabolic pathways (Fig. 4j, k). These subtypes are associated with multiple pro-tumor metabolic pathways, such as glycolysis, the citrate cycle (TCA cycle)^103,104^, the pentose phosphate pathway^105^, pyruvate metabolism^106^, fatty acid biosynthesis^107^, glutathione metabolism^108^, and glycine, serine^109^, and threonine metabolism^110^ (Supplementary Fig. S12). Therefore, targeting TAM metabolic reprogramming represents a promising strategy for tumor therapy.

### Complex regulatory networks between TAMs and CAFs create a favorable microenvironment for tumor survival and metastasis

**Fig. 7:**
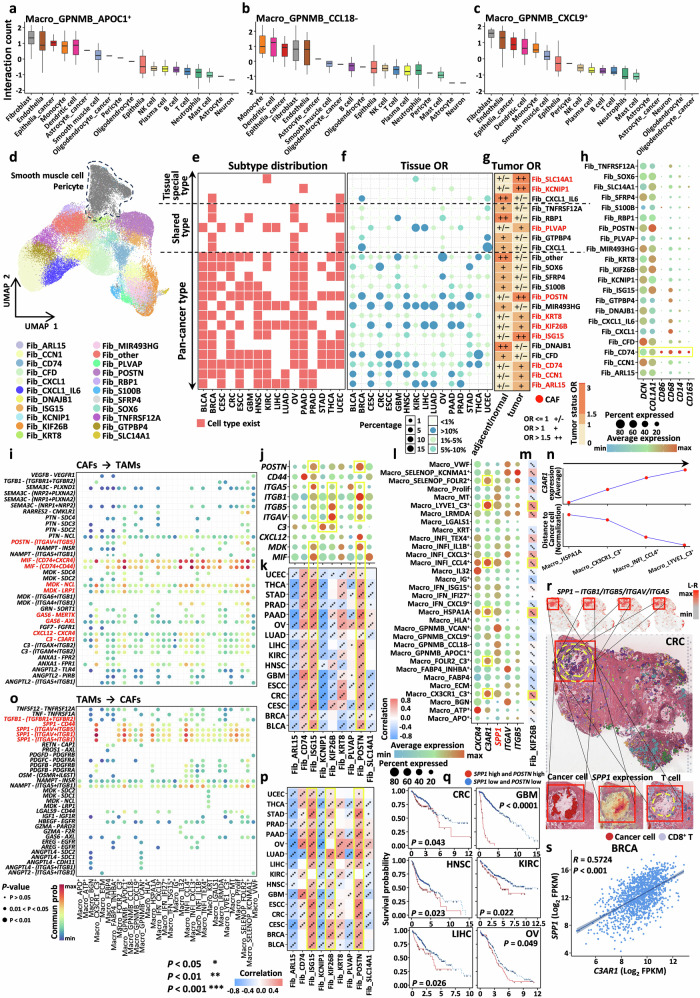
Complex regulatory networks between TAMs and CAFs create a favorable microenvironment for tumor survival and metastasis. **a–c** Boxplots showing the interaction strength between different TAM subtypes and various components of the TME. **d** UMAP plot showing the integration results of fibroblasts across different cancer types. **e** Heatmap depicting the fibroblast subtypes present across different cancer types. **f** Bubble plot illustrating the relative proportions of different fibroblast subtypes across cancer types, with both bubble size and color encoding the magnitude of the proportion. **g** Heatmap showing the preference of different fibroblast subtypes in tumor tissues, where values greater than 1 indicate significant tissue preference. **h** Bubble plot displaying the expression of macrophage signature genes in different fibroblast subtypes. **i** Bubble plot showing the interaction relationships between CAFs and TAMs, along with ligand-receptor pairs. **j** Bubble plot showing the expression of key receptors and ligands in CAFs based on CellChat analysis. **k** Heatmap showing the correlation between CAF abundance and the M2 score (*P < 0.05, **P < 0.01, ***P < 0.001). **l** Bubble plot showing the expression of key receptors and ligands in CAFs based on CellChat analysis. **m** Heatmap showing the correlation between the abundance of the ligand *C3AR1* and that of Fib_KIF26B cells (*P < 0.05, **P < 0.01, ***P < 0.001). **n** Dot plot showing the distance between four key TAM subtypes and cancer cells, as well as the average expression of *C3AR1* in these subtypes. **o** Bubble plot showing the interaction relationships between TAMs and CAFs, along with ligand-receptor pairs. **p** Heatmap showing the correlation between *SPP1* expression and CAF abundance (*P < 0.05, **P < 0.01, ***P < 0.001). **q** Kaplan‒Meier curves showing the association between the co-expression of *SPP1* and *POSTN* and prognosis, with *P* values calculated using the log-rank test. **r** Top: co-expression of *SPP1* and integrin receptors (*ITGB1*/*ITGB5*/*ITGAV*/*ITGA5*) on spatial slices. Middle: spatial distribution of key TAMs, CAFs, and CD8^+^ T cells visualized using CellTrek. Bottom: spatial distribution of cancer cells and T cells and expression of *SPP1* on spatial slices visualized using CellTrek. **s** Correlation between *SPP1* and *C3AR1* expression.

CAFs are among the most critical and abundant stromal cells within the TME and play pivotal roles in processes such as angiogenesis, extracellular matrix remodeling, and immune evasion^1,111,112^, making them a subject of significant research interest. Through cell‒cell interaction analysis, we detected strong interactions between TAMs and CAFs (Figs. 1i, 7a–c and Supplementary Fig. S13, S14a). Therefore, delving into the mechanism underlying the interactions between TAMs and CAFs is particularly important.

Currently, the classification of fibroblast subtypes at the single-cell level has begun to take shape^34,113,114^. We integrated fibroblasts from all the samples and performed reclustering. Using a similar analytical approach, we defined a total of 22 subtypes. In accordance with the methodology used for macrophages, we categorized these subtypes into pan-cancer subtypes, shared subtypes, and tissue-specific subtypes based on their prevalence across different tissue types (Fig. 7d–f and Supplementary Fig. S14b–e). Additionally, drawing on definitions from previous literature^34^, we identified subtypes enriched in tumor tissues as CAFs (Fig. 7g). In subsequent analyses, we focused on the critical roles of these CAFs.

Interestingly, we identified a CAF subtype, Fib_CD74, characterized by high expression of monocyte/macrophage-specific markers (e.g., *CD68*, *CD14*, and *CD163*) (Fig. 7h). This subtype has been previously reported in the literature as an antigen-presenting CAF subtype^34^ (Supplementary Fig. S14f). Stromal cells such as tissue-resident fibroblasts, endothelial cells, and pericytes are major sources of CAFs. However, numerous studies have also suggested that macrophages can serve as important sources of CAFs, with their transformation playing a regulatory role in pathological processes such as renal fibrosis^115^ and myocardial infarction healing^116^. Within our TAM population, we identified subtypes (Macro_BGN, Macro_ECM, and Macro_VWF) that highly expressed genes involved in extracellular matrix remodeling and were spatially enriched around fibroblasts (Fig. 4j and Supplementary Fig. S7c, e, S8o). Previous analyses and reports have linked these subtypes to resistance to ICI therapy (Supplementary Fig. S9h). We hypothesized that these three TAM subtypes and Fib_CD74 represent transitional states on a developmental continuum. To test this hypothesis, we performed pseudotime trajectory analysis. The results revealed that, except for Macro_BGN, Macro_ECM, Macro_VWF and Fib_CD74 span the entire developmental axis from macrophages to CAFs, with a particular concentration in the transitional region (Supplementary Fig. S14g–i). Further enrichment analysis indicated that the transitional region involves the activation of pathways such as TNFα signaling via NF-κB, KRAS, TGFβ, EMT, MYC, and angiogenesis (Supplementary Fig. S14j), suggesting that this transformation process may also be linked to cancer progression.

To comprehensively characterize the cellular communication between different TAM subtypes and CAFs, we integrated various CAF subtypes and performed cell-cell interaction analysis using CellChat software. We first focused on the regulatory effects of CAFs on TAMs and observed that the *MIF*-(*CD74* + *CXCR4*/*CD44*) and *MDK*-(*NCL*/*LRP1*) signaling axes were prominently involved in these regulatory interactions (Fig. 7i). As shown above, we found that cancer cells could regulate M2 macrophage polarization through these two axes (Fig. 6). Further analysis revealed that *MDK* and *MIF* were specifically highly expressed in Fib_ISG15 and Fib_POSTN cells (Fig. 7j), and the abundance of these two subtypes was significantly positively correlated with the M2 score of macrophages at the pan-cancer level (Fig. 7k). We identified these subtypes as highly similar to myofibroblasts (Supplementary Fig. S14f), suggesting that myofibroblasts are also a significant source of *MIF* and *MDK* and play a role in regulating macrophage polarization. Additionally, we found that CAFs promoted TAM survival and polarization through the *GAS6*-*MERTK*/*AXL* interaction axis (Fig. 7i), with the *MERTK* and *AXL* receptor families, which are highly expressed on TAMs, being considered promising therapeutic targets^117^. We also focused on another widely observed interaction axis, *C3*-*C3AR1*. Recently, the non-canonical functions of complement *C3*, primarily mediated through its homologous membrane-bound receptor *C3AR1*, have been confirmed (Supplementary Fig. S15a)^118^. *C3* is predominantly expressed in Fib_KCNIP1 and Fib_KIF26B cells, with Fib_KIF26B cells accounting for a significant proportion of fibroblasts (Fig. 7f; Supplementary Fig. S14e). In contrast, *C3AR1* is consistently expressed in a subset of TAMs that exhibit tissue-resident macrophage characteristics (Fig. 7l). Correlation analysis demonstrated that the Fib_KIF26B subtype was strongly associated with four macrophage subtypes: Macro_CX3CR1_C3^+^, Macro_HSPA1A, Macro_INFI_CCL4^+^, and Macro_LYVE1_C3^+^ (Fig. 7m). Notably, these four subtypes were highly enriched in adjacent normal or healthy tissues (Fig. 2e). Spatial analysis further confirmed that the expression of *C3AR1* was negatively correlated with the distance of these cells from cancer cells (Fig. 7n). These findings suggest that Fib_KIF26B may recruit macrophages from normal tissues to tumor-adjacent regions through the *C3*-*C3AR1* axis.

In our subsequent investigation of the TAM-mediated regulation of CAFs, we identified the *SPP1*-*CD44* and *SPP1*-(*ITGAV*/*ITGA5*/*ITGB1*/*ITGB5*) axes as the most prominent interaction pathways (Fig. 7o). In our dataset, we observed that *SPP1* is widely expressed across multiple TAM subtypes (Fig. 7l), indicating that the broad cellular sources of *SPP1*-*CD44*, a key molecule in fibroblast activation, can also increase *CXCL12* expression through ERK phosphorylation^119,120^. The *CXCL12*-*CXCR4* interaction axis has been shown to promote the survival and lymphatic metastasis of malignant epithelial cells, as demonstrated in oral cancer^121^. Additionally, a previous pan-cancer study revealed that TAMs and CAFs can promote intratumoral angiogenesis through the *SPP1*-*CD44* interaction^34^.

We further conducted correlation analysis, which revealed that the expression of *SPP1* was most strongly correlated with the Fib_ISG15 and Fib_POSTN subtypes (Fig. 7p). Both Fib_ISG15 and Fib_POSTN co-expressed the characteristic gene *POSTN* (Fig. 7j). *POSTN*, a key marker of CAFs, has been reported to be involved in epithelial cell adhesion and migration, as well as the maintenance and metastasis of cancer stem cells^122–124^. Prognostic analysis confirmed that the co-high expression of *SPP1* and *POSTN* is associated with poor patient outcomes across multiple cancer types, suggesting that the interaction between *SPP1* and Fib_ISG15/Fib_POSTN cells may serve as a critical marker for tumor progression (Fig. 7q and Supplementary Fig. S16). The receptors of *SPP1* (*ITGAV*, *ITGA5*, *ITGB1*, and *ITGB5*) are members of the integrin family and are known for their roles in promoting stromal cell adhesion and migration. Therefore, we hypothesized that *SPP1* secreted by TAMs enhances the adhesion of myofibroblasts around tumors, thereby facilitating tumor invasion and metastasis. This hypothesis was supported by spatial transcriptomic data, which revealed that *SPP1* and integrin family receptors were co-expressed in tumor regions, whereas Fib_ISG15 and Fib_POSTN myofibroblasts were clustered around cancer cells (Fig. 7r). CD8^+^ T cells were found to be sequestered around myofibroblasts, limiting their infiltration into the tumor core, which may contribute to the formation of an immunosuppressive tumor microenvironment. In previous studies, *SPP1* was confirmed to induce fibroblast activation and proliferation by binding to *ITGAV* and *ITGB1*^113^. Surprisingly, *C3AR1*, which was identified in previous analyses, appeared to increase *SPP1* expression, with a strong positive correlation across multiple cancer types (Fig. 7s and Supplementary Fig. S15b). Further analysis revealed that Fib_KIF26B, a CAF subtype highly expressing *C3* (the ligand of *C3AR1*), also showed a significant positive correlation with *SPP1* levels (Fig. 7r). We speculate that, in addition to recruiting macrophages from normal tissues, Fib_KIF26B cells may also increase the expression of *SPP1* in TAMs. The results of the above comprehensive analysis highlight the critical regulatory role of *SPP1*, an important secretory protein of TAMs, in CAFs. These interactions create favorable conditions for further tumor progression.

## Discussion

By integrating cross-tissue scRNA-seq and ST data from 16 common cancer types, we comprehensively characterized the biological properties of TAMs within the TME (Fig. 1). We identified 28 TAM subtypes, including 21 pan-cancer subtypes as well as several previously unrecognized or underappreciated states (e.g., Macro_LGALS1 and Macro_IL32). These subtypes were systematically profiled in terms of their biological functions, lineage relationships, clinical relevance, and spatial distribution patterns. Notably, despite the heterogeneous origins of the datasets, they were successfully harmonized within a unified analytical framework, providing a robust reference resource for future studies (Figs. 2, 3). Nevertheless, it should be acknowledged that current computational constraints limit our ability to capture the full spectrum of dynamic macrophage states across all tissue contexts, such as inflammatory tissues, metastatic lesions, and peripheral blood. Importantly, this limitation does not detract from the depth and rigor of our analyses within the TME.

Using ST data from multiple tissue slices, we meticulously characterized the spatial distribution patterns of TAMs within the TME. At the pan-cancer level, different TAM subtypes maintain close spatial relationships with one another. Monocytes, as a key source of TAMs, are enriched in regions surrounding TAMs. In contrast, most TAMs maintain a considerable spatial distance from other immune cells, which may contribute to the formation of an immunosuppressive microenvironment. Additionally, we found that the spatial localization and developmental trajectories of TAMs within the TME are closely linked to the activation of their specific biological functions (Fig. 4).

We systematically investigated the crosstalk between TAMs and TME components during tumor progression. We identified Macro_GPNMB_VCAN^+^, a TAM subtype enriched in hypoxic and necrotic tumor core regions, as the macrophage population most strongly interacting with cancer cells. This subtype promotes tumor progression via the *SPP1/LGALS9*-*CD44* signaling axis and is tightly linked to metabolic reprogramming, particularly glycolysis and lactate production. Beyond this subtype, we demonstrate that TAMs broadly participate in tumor metabolic reprogramming, with pronounced activity in the tumor core across key pathways, including glycolysis, the TCA cycle, the pentose phosphate pathway, and pyruvate metabolism. These findings highlight metabolically specialized TAM subpopulations as promising therapeutic targets for disrupting tumor progression (Fig. 6).

Additionally, we identified an inflammatory TAM subtype, Macro_INFI_IL1B^+^, that engages in extensive crosstalk with IFN^+^ macrophage subtypes. Notably, IFN^+^ Macro subtypes, particularly Macro_IFN_CXCL9^+^ and Macro_IFN_ISG15^+^, which specifically overexpress immune checkpoint-related genes (e.g., *CD274*), are enriched in immunotherapy-responsive patients and actively recruit CD8⁺ T cells to the TME, highlighting their relevance to immunotherapy. By integrating our data with those of previous studies, we revealed a previously underappreciated macrophage-macrophage-T-cell interaction axis, whereby IFN^+^ macrophages retain CD8⁺ T cells at the tumor periphery, inducing localized inflammation and activating the *NLRP3* inflammasome in Macro_INFI_IL1B^+^ cells. This activation enhances *IL1B* and *CXCL8* secretion, driving neutrophil recruitment, angiogenesis, and tumor invasion in the tumor core. In parallel, Macro_INFI_IL1B^+^ cells directly promote tumor progression via the *EREG*/*AREG*-*(EGFR/ERBB2)* signaling axis. Together, these findings position Macro_INFI_IL1B^+^ cells as a key inflammatory and pro-tumorigenic hub in the TME, warranting further investigation as a potential therapeutic target (Figs. 5, 6).

CAFs, the most abundant stromal cells in the TME, are also the cell population that most closely interacts with TAMs, making their crosstalk particularly critical for tumor progression. We constructed a bidirectional and dynamic TAM-CAF interaction network with several key features. First, TAMs can serve as a cellular source of CAFs, with intermediate states exhibiting both macrophage traits and extracellular matrix–remodeling programs, accompanied by the activation of EMT- and TGF-β–related pathways. Second, the pan-cancer CAF subtype Fib_KIF26B recruits macrophages from normal tissues via the *C3*-*C3AR1* axis and promotes their conversion into tumor-supportive TAMs. Third, we identified *SPP1* as a central mediator of TAM-CAF crosstalk: most TAM subtypes can secrete *SPP1*, which activates fibroblasts through *CD44* and integrin receptors, promotes angiogenesis, and enhances *CXCL12* expression in CAFs, thereby promoting the activity of the *CXCL12*-*CXCR4* axis in malignant epithelial cells. Notably, the *POSTN*-high CAF subtypes are strongly correlated with *SPP1* expression, and their co-expression is associated with a poor prognosis across multiple cancers. Spatial analyses further confirmed the co-localization of *SPP1* and POSTN in tumor-enriched regions, where *POSTN*-high CAFs facilitate tumor invasion while forming physical barriers that restrict T-cell infiltration and reinforce immunosuppression. Collectively, our findings define a TAM–CAF interaction axis centered on *SPP1* that integrates stromal remodeling, immune exclusion, and tumor progression, highlighting a promising therapeutic target in the TME (Fig. 7).

Finally, the binary M1/M2 polarization of macrophages is a process that spans most of their developmental stages and is associated with various pathological mechanisms, including cancer. Although some subtypes exhibit polarization preferences, most TAMs often co-express both M1 and M2 signature genes, indicating that TAMs frequently possess characteristics of both M1 and M2 phenotypes (Fig. 2). We also identified cancer cells and myofibroblasts as significant sources of *MIF* and *MDK* ligands, which can induce M2 macrophage polarization through the *MIF*-(*CD74* + *CXCR4*/*CD44*) and *MDK*-(*NCL*/*LRP1*) interaction axes. Preventing the M2 polarization of TAMs has become a key strategy in tumor therapy, and whether these interaction axes can serve as critical markers for reversing M2 polarization warrants further investigation (Figs. 6, 7).

However, this study has several limitations. First, the lack of matched scRNA-seq and ST datasets may introduce bias in spatial cell-type annotation. Future studies will incorporate additional matched samples to further expand and refine the current multi-omics data resources. Second, owing to practical challenges in obtaining high-quality clinical specimens, experimental validation of key TAM subsets at a pan-cancer scale was not feasible in the present study. Subsequent work will involve the collection of high-quality clinical tissue samples, combined with multiplex immunofluorescence or spatial proteomic approaches, to validate key TAM subtypes and their spatial co-localization patterns. Third, cell-cell communication analyses are constrained by existing computational frameworks, and inferences regarding interactions between TAMs and other components of the tumor microenvironment are primarily based on computational analyses. Future studies will therefore integrate in vitro functional assays and animal models to systematically evaluate the roles of key molecular interaction axes in regulating immunotherapy responses and tumor metastasis.

In summary, in this study, a pan-cancer atlas of TAM heterogeneity was constructed to comprehensively delineate the functional diversity and spatial organization of distinct TAM subtypes across multiple tumor types. Building upon this framework, we further elucidated TAM-centered cellular communication networks and highlighted the critical roles of coordinated interactions between TAMs and diverse components of the tumor microenvironment in shaping tumor progression and immune regulation. Accumulating evidence indicates that TAMs are not merely passive immune infiltrates but rather function as key regulatory hubs within the tumor microenvironment and represent highly actionable therapeutic targets. Current strategies aimed at remodeling the tumor microenvironment primarily involve inhibiting TAM recruitment and survival, reprogramming immunosuppressive TAM phenotypes toward anti-tumor states, and disrupting pro-tumorigenic interactions between TAMs and stromal or immune cells, frequently in combination with immune checkpoint blockade therapies^125–127^. In this context, our pan-cancer analyses revealed that multiple TAM-associated molecular programs and interaction patterns are highly conserved across tumor types, providing a system-level framework for understanding TAM-mediated immune modulation. Guided by the multi-omics insights generated in this study, future work will integrate in vitro and in vivo functional investigations to systematically validate the biological roles of key TAM subpopulations and their core molecular interaction axes, thereby facilitating mechanistic dissection and promoting the translational development of TAM-targeted therapeutic strategies.

## Methods

### Dataset collection and integration

The scRNA-seq datasets for the 16 cancer types used in this study were obtained from the GEO database (https://www.ncbi.nlm.nih.gov/geo/), encompassing 212 samples from 17 studies. Sample information was extracted from the corresponding studies (Supplementary Table S1). The ST datasets for 14 cancer types were sourced from the GEO and CROST databases (https://ngdc.cncb.ac.cn/crost/), comprising 79 samples from 17 studies, with sample information also derived from the respective studies (Supplementary Table S2). Bulk RNA-seq datasets for 16 cancer types and their corresponding adjacent normal or healthy tissues were obtained from the TCGA (https://www.cancer.gov/ccg/research/genome-sequencing/tcga) and GTEx (https://www.gtexportal.org/) databases, with sample and survival prognosis information retrieved from the platform-curated data (Supplementary Tables S3 and S4). TMB data were downloaded as processed datasets from the cBioPortal database (https://www.cbioportal.org/) (Supplementary Table S5). Samples receiving ICI treatment were sourced from 16 studies, with all sample characteristics and survival prognosis information obtained from the original publications (Supplementary Table S10).

### scRNA-seq data processing

The collected 10x Genomics FASTQ files were aligned and quantified against the GRCh38 human reference genome using Cell Ranger software (version 7.2.0) with default settings. The count matrices processed by Cell Ranger were read using the Read10X function from the Seurat package (version 5.0.2)^128^. Additionally, quality control of the cells was performed based on several criteria. Briefly, cells whose total gene count was less than 500 or whose mitochondrial content exceeded 15% were removed. Cells with gene counts exceeding 5000 or UMI counts greater than 40,000 were eliminated to exclude doublets. After filtering, a total of 1,039,479 high-quality cells were retained for subsequent analysis. The global scaling normalization method (“LogNormalize”) was applied to ensure equal total gene expression per cell, with a scale factor set to 10,000. The FindVariableFeatures function was used to identify the top 3000 most highly expressed genes for downstream analysis. The ScaleData function, with the “vars.to.regress” option for UMI and mitochondrial content percentage, was employed to regress out unwanted sources of variation. Principal component analysis (PCA) based on highly variable features was performed to reduce the dimensionality of the dataset, with the number of PCs determined by the elbow plot generated using the ElbowPlot function. To mitigate batch effects, the RunHarmony function from the Harmony package (version 1.2.0) was used for batch correction^26^. The compute_lisi function from the lisi package (version 1.0.0) was applied to calculate the Local Inverse Simpson’s Index (LISI) before and after batch effect removal to assess the effectiveness of batch correction. An LISI greater than 1 for all cells indicated no significant batch effects. Clustering analysis was conducted based on the edge weights between any two cells, and the Louvain algorithm, implemented in the FindNeighbors and FindClusters functions, was used to generate a shared nearest neighbor graph. The identified clusters were visualized using the uniform manifold approximation and projection (UMAP) method. To annotate cell clusters, the FindAllMarkers function with the default non-parametric Wilcoxon rank-sum test and Bonferroni correction was used to identify differentially expressed markers for each cluster. The DotPlot function was employed to visualize the expression of specific genes.

### Assessment of TAMs for cross-organizational data integration

To systematically evaluate myeloid cell integration across tissues, we employed the scIB framework for comprehensive assessment across ten metrics. These metrics were categorized into two key dimensions: biological conservation (preserving true biological variation) and batch correction (removing technical artifacts). A composite score derived from both dimensions served as our final evaluation metric, with higher scores indicating superior integration performance. We used the scib_metrics package (version 0.5.1)^32^ to complete the above analysis.

To assess whether our clustering effectively captured the transcriptional heterogeneity of TAMs and to determine whether further subclustering was warranted, we applied the ROGUE metric, an entropy-based measure of population purity. ROGUE values approaching 1 indicate highly homogeneous cell subsets with minimal intra-cluster transcriptional variation. We used the rogue function of the ROGUE package (version 1.0.0)^33^ to complete the above analysis.

### Spatial transcriptomic data processing

For ST datasets with raw data available, we used the Space Ranger software (version 2.1.1) with default settings to align and quantify the collected 10x Visium FASTQ files against the GRCh38 human reference genome. The processed and publicly available ST datasets were read using the Load10X_Spatial function from the Seurat package. SCTransform was applied to ensure equal total gene expression per spot, with a scale factor set to 12,000. For ST datasets, no integration analysis was performed, and a total of 236,716 spots were defined.

### Bulk RNA-seq data processing (TCGA and GTEx)

To eliminate data bias caused by differences in processing pipelines, we applied the Toil RNA-seq pipeline from the UCSC Xena database (https://xena.ucsc.edu/) to quantify gene expression levels in TCGA and GTEx samples; hg38 was used as the reference genome, and RSEM was used to quantify gene expression. Gene expression was quantified using FPKM, and the final expression profiles were log_2_(FPKM + 0.001) transformed. Healthy tissue data from the same anatomical sites were matched to the corresponding cancer datasets.

### Bulk RNA-seq data processing (ICI-treated samples)

We integrated bulk RNA-seq datasets from ICI-treated samples across various database platforms and studies, primarily including samples from immunotherapy responders and non-responders. Owing to significant differences in sample sources or sequencing methods, we first normalized the data using the z-score function and then applied the removeBatchEffect function from the limma package (version 3.56.2)^129^ to eliminate batch effects between datasets from different sources, ensuring that all samples were on a unified scale.

### Recognition of malignant and nonmalignant cells

The copykat function from the CopyKAT package (version 1.1.0)^27^ was used with default parameters to identify malignant and non-malignant cells. Our input files included the raw count matrix extracted from the Seurat object and the cell type annotation file, with T cells, B cells, or NK cells from the samples serving as normal controls. Epithelial or glial cells with malignant tendencies across all the samples were defined as cancer cells.

### CellTrek spatial deconvolution analysis

To obtain spatial coordinates for individual cells, we performed an integrated analysis of the scRNA-seq and ST data using the CellTrek package (version 0.0.94)^31^. First, the Seurat object was converted into a format suitable for CellTrek using the traint function, with parameters specified as st_assay = “spatial”, sc_assay = “RNA”, norm = “LogNormalize”, and nfeatures = 3000. The celltrek function was subsequently used to infer the spatial locations of individual cells, with parameters set as int_assay = “traint”, sc_assay = “RNA”, reduction = “pca”, intp = T, intp_pnt = 10,000, and intp_lin = F. Finally, the kdist function was employed to infer spatial distances between individual cells, with parameters specified as ref_type = “all”, keep_nn = F and *k* = 10.

### Spatial distribution pattern inference

We used the kdist function from the CellTrek package to calculate the spatial distances between different cells across all ST slices. To determine the pan-cancer spatial distances between all TAM subtypes and other cell types, we extracted the spatial distances of the specified cell types from all the other cell types on each slice and then calculated the average distances across the 79 slices. To infer the characteristics of the microenvironment surrounding the TAMs, we calculated the proportions of different cells within and beyond 100 μm of the spatial distance from specific cell types. Finally, paired *t*-tests were used to assess the differences in cell type abundance between the proximal and distal regions.

### Preference analysis

To assess the cancer or tissue preferences of different cell types, the odds ratio (OR) was calculated using the computational method described by Zhang et al. This involved constructing a 2 × 2 contingency table for each combination of cell type i and cancer type j. The table included the number of cells from cell type i in cancer type j, the number of cells from cell type i in other cancer types, the number of cells from non-i cell types in cancer type j, and the number of cells from non-i cell types in other cancer types. Fisher’s exact test was then performed on the contingency table.

### Cell-cell interaction analysis

We conducted interaction analysis using the CellChat package (version 1.1.3)^28^. The Seurat object was converted into a CellChat object using the createCellChat function, with cell_type specified as the grouping information. The built-in ligand-receptor database CellChatDB.human was used as the foundation for the analysis. The computeCommunProb and filterCommunication functions were employed to infer potential ligand-receptor interactions between different cell types. Finally, the netVisual_bubble function was used to visualize the interaction patterns of signaling pathways and ligand-receptor pairs among different cell types.

### NicheNet analysis

To further identify the key mediators between the IFN^+^ Macro and CD8^+^ T-cell subsets, we employed the NicheNet software package (version 2.1.5)^70^ to infer their interactions. For ligand-receptor interactions, genes expressed in more than 10% of the cell clusters were considered. The top 30 ligands from the differentially expressed genes of the “sender cells” and “receiver cells” were extracted for paired ligand-receptor activity analysis. We used the active_ligand_target_links function to calculate the potential regulatory strength between ligands and targets. The expression of differentially expressed ligands and receptors was also visualized in heatmaps by computing the average gene expression within specified cell types, with scaling applied across specified subtypes. The make_heatmap_ggplot function was utilized for visualizing ligand activity and interaction strength heatmaps.

### Functional enrichment analysis

Functional enrichment analysis was performed using the enricher function from the clusterProfiler package (version 3.0.4)^130^ for GO biological functions, KEGG pathways and hallmark gene sets, with the parameter set to pvalueCutoff = 0.05. To infer the functional characteristics of macrophages, we conducted enrichment analysis using signature genes of macrophage subtypes obtained from differential expression analysis across different cancer datasets. To systematically identify aberrantly activated pathways of macrophages at the pan-cancer level, we first performed pathway enrichment analyses based on differentially expressed genes for each cancer type and selected the top 30 pathways with the greatest statistical significance. We then integrated these results across cancer types, quantified the top 15 pathways that occurred most frequently among the 16 cancers, and visualized them using a heatmap to delineate shared pan-cancer features. The remaining enrichment results were ranked according to their significance and are displayed as bar plots to further illustrate the relative importance of different pathways.

The GSVA package (version 1.14.1)^131^ was used with the enrichment method set to ssGSEA to infer the association between different TAM subtypes and hallmark gene sets. Gene expression levels for different cells were obtained using the AverageExpression function.

All enrichment pathways were sourced from the MSigDB database (https://www.gsea-msigdb.org/gsea/msigdb)^132^.

### Single-cell developmental trajectory analysis

To infer the overall developmental trajectory of TAMs, we applied the Monocle algorithm (version 2.28.0). The newCellDataSet function was used to create a new Monocle object using transcript count data from the included cell populations. The results generated by the estimateSizeFactors and estimateDispersions functions helped normalize the differences in mRNA recovery between cells. The feature genes expressed in at least 10% of the cells in the dataset and whose *P*-value was < 0.01 according to the differentialGeneTest function were included to define the trajectory progression. The reduceDimension function reduced the space to two dimensions, and the orderCells function ordered cells based on gene expression. Pseudotime-dependent genes were calculated using the differentialGeneTest function with the fullModelFormulaStr option set to ~ sm.ns(Pseudotime), and smooth expression curves were generated using the plot_pseudotime_heatmap function. Enrichment analysis for different clusters along the time series was performed using the compareCluster function with the enrichment method set to enrichGO.

### Inference of TAM subtype abundance in bulk RNA-seq data using ssGSEA

To infer the abundance of TAM subtypes in the TCGA, GTEx and integrated bulk RNA-seq datasets from ICI-treated samples, we used signature genes of different subtypes (filtered by FC ≥ 1.5 and pct.1 ≥ 0.5) and applied the ssGSEA algorithm implemented in the GSVA package (version 1.14.1)^131^.

### xCell inference of cell proportions in bulk RNA-seq data

The xCell package (version 1.1.0)^64^ was used to infer the proportions of different cell types in the bulk RNA-seq samples. We employed the xCellAnalysis function with default parameters to estimate the proportions of various cell types in the TCGA and GTEx samples.

### Quantification of transcriptomic similarity and co-occurrence patterns across TAM subtypes

To assess the transcriptomic similarity of the 28 TAM subtypes identified in this study, we performed unsupervised hierarchical clustering analysis based on a Euclidean distance matrix to generate a dendrogram. Additionally, we inferred co-occurrence patterns between TAM subtypes and between TAMs and other TME components in different tissue groups (tumor tissues and adjacent normal or healthy tissues). To determine the co-occurrence patterns between the TAM subtypes, we conducted sample-level Pearson correlation analysis using the abundance inferred from the bulk RNA-seq data by the ssGSEA algorithm. To determine the co-occurrence patterns between TAMs and other TME components, we performed sample-level Pearson correlation analysis using the abundance of bulk RNA-seq data inferred by both the ssGSEA and xCell algorithms. Both positive and negative correlations were considered.

### Spatial developmental trajectory analysis

We first utilized the RCTD algorithm from the spacexr software package (version 2.2.1)^66^ to infer the proportion of different cell types within each spot on the spatial transcriptomic slices. We employed the create.RCTD function to convert the Seurat object into the format required by RCTD. Subsequently, the run.RCTD function was used to infer the proportions of cell types across different spots. Finally, the normalize_weights function was applied to normalize the proportions within each spot, and the results were integrated into the corresponding Seurat object. After excluding spots with TAM proportions less than 5%, we applied the Monocle2 algorithm (version 2.28.0) to infer the developmental trajectories of the retained spots. Finally, we performed enrichment analysis on clusters along the pseudotime trajectory using the compareCluster function, with the enrichment method set to enrichGO.

### Spatial gene co-expression analysis

We used the SpaGene software package (version 0.1.0)^133^ to visualize the co-expression patterns of specific gene pairs on spatial slices. To infer the co-expression patterns of specific ligand-receptor pairs on spatial slices, ST expression profiles were obtained using the GetAssayData function, followed by the SpaGene function to infer spatial co-expression levels. Finally, the plotLR function was used to visualize the co-expression levels of specific ligand-receptor pairs.

### Single-cell metabolic activity analysis

To evaluate the metabolic pathway activity of Macro_GPNMB_VCAN^+^ cells, we utilized the SCPA software package (version 1.6.2)^87^ to analyze the pan-cancer single-cell dataset for Macro_GPNMB_VCAN^+^ cells, employing metabolic pathway gene sets obtained from the supplementary materials of Bibby et al. (Supplementary Table S12).

Additionally, we employed the scMetabolism software package (version 0.2.1)^102^ to conduct metabolic pathway analysis across different TAM subtypes. The function sc.metabolism. Seurat (method = “AUCell”) was used with default parameters to quantify the metabolic activity of various TAM subtypes. The results were visualized using the DotPlot.metabolism function.

### Survival analysis

Survival analysis was performed using the R survival package (version 3.5.7). A Cox proportional hazards model was used to calculate hazard ratios (HRs) and 95% confidence intervals (CIs). The survfit function was used to construct Kaplan‒Meier survival curves, and the log-rank test (two-sided) was applied to compare survival differences.

To assess the relationship between macrophage abundance and prognosis, samples in the top 25% of abundance were defined as high-abundance, while those in the bottom 25% were defined as low-abundance.

To determine the relationship between the abundance of FOLR2^+^ Macro and CD8^+^ T cells and prognosis, samples where the abundance of both FOLR2^+^ Macro and CD8^+^ T cells was above the median were defined as having high expression, whereas those where the abundance of both subtypes was below the median were defined as having low expression.

With respect to the relationship between *SPP1* and *POSTN* coexpression and prognosis, samples with both *SPP1* and *POSTN* expression levels above the median were defined as having high expression, whereas those with both expression levels below the median were defined as having low expression.

### Statistical analysis

All the statistical analyses were performed using R software (version 4.1.0). Differences between two groups were analyzed using the Wilcoxon test. Survival curves depicted by Kaplan‒Meier curves were compared using the log-rank test. Statistical differences between groups were assessed using the Wilcoxon test, whereas paired *t*-tests were employed to compare the differences in abundance between proximal and distal cells at the spatial level. Correlation coefficients were calculated using Pearson’s method. A *P*-value < 0.05 was considered to indicate statistical significance. Significance levels are denoted as follows: *P < 0.05, **P < 0.01, and ***P < 0.001.

## Supplementary information

Supplementary Figures

Supplementary Tables

## Data Availability

This study did not generate new datasets. All the original data were obtained from public databases, including GEO, TCGA, GTEx, CROST, and cBioPortal. Detailed sample information can be found in Supplementary Tables S1, S2, S3, S4, S5, and S10. The scRNA-seq atlas (TAM atlas) generated in this study has been deposited in Zenodo as a Seurat object under accession code 14966060. This study does not report the original code.

## Code availability

This study did not generate new datasets. All the original data were obtained from public databases, including GEO, TCGA, GTEx, CROST, and cBioPortal. Detailed sample information can be found in Supplementary Tables S1, S2, S3, S4, S5, and S10. The scRNA-seq atlas (TAM atlas) generated in this study has been deposited in Zenodo as a Seurat object under accession code 14966060. This study does not report the original code.

## Materials availability

This study did not generate new unique reagents.
