# Supplementary material for: Spatial single-cell landscape of tumor-associated macrophages and their crosstalk with the tumor microenvironment
Source: Cell Discov. 2026 May 19;12:35. doi: 10.1038/s41421-026-00888-3 (PMC13187174; doi:10.1038/s41421-026-00888-3)
Supplement: Supplementary file 1 — Supplementary Figures [file 41421_2026_888_MOESM1_ESM.pdf]

Spatial single-cell landscape of tumor-associated macrophages and their crosstalk with the tumor microenvironment

Rui-chao Nie [1, 2, 3, #], Guo-sheng Hu [5, #], Shi-qiang Cao [6, #],
An Wang[2, 3], Du-chuang Wang [2, 3], Wen Liu[1, 2, 3, 4] *

[1]National Institute for Data Science in Health and Medicine,
Xiamen University, Xiang'an South Road, Xiamen, Fujian, China.
[2]State Key Laboratory of Vaccines for Infectious Diseases, Xiang An Biomedicine Laboratory,
School of Pharmaceutical Sciences, Faculty of Medicine and Life Sciences,
Xiamen University, Xiang'an South Road, Xiamen, Fujian, China.
[3]Fujian Provincial Key Laboratory of Innovative Drug Target Research,
School of Pharmaceutical Sciences, Faculty of Medicine and Life Sciences,
Xiamen University, Xiang'an South Road, Xiamen, Fujian, China.
[4]Shenzhen Research Institute of Xiamen University,
Shenzhen, Guangdong, China.
[5]Biomedical Research Center of South China, College of Life Sciences, Fujian Normal University, Fuzhou, Fujian, China.
[6]Department of Thoracic Surgery, Fujian Medical University Union Hospital, No. 29 Xinquan Road, Fuzhou, Fujian, China.

[#] These authors contributed equally
* Correspondence: w2liu@xmu.edu.cn

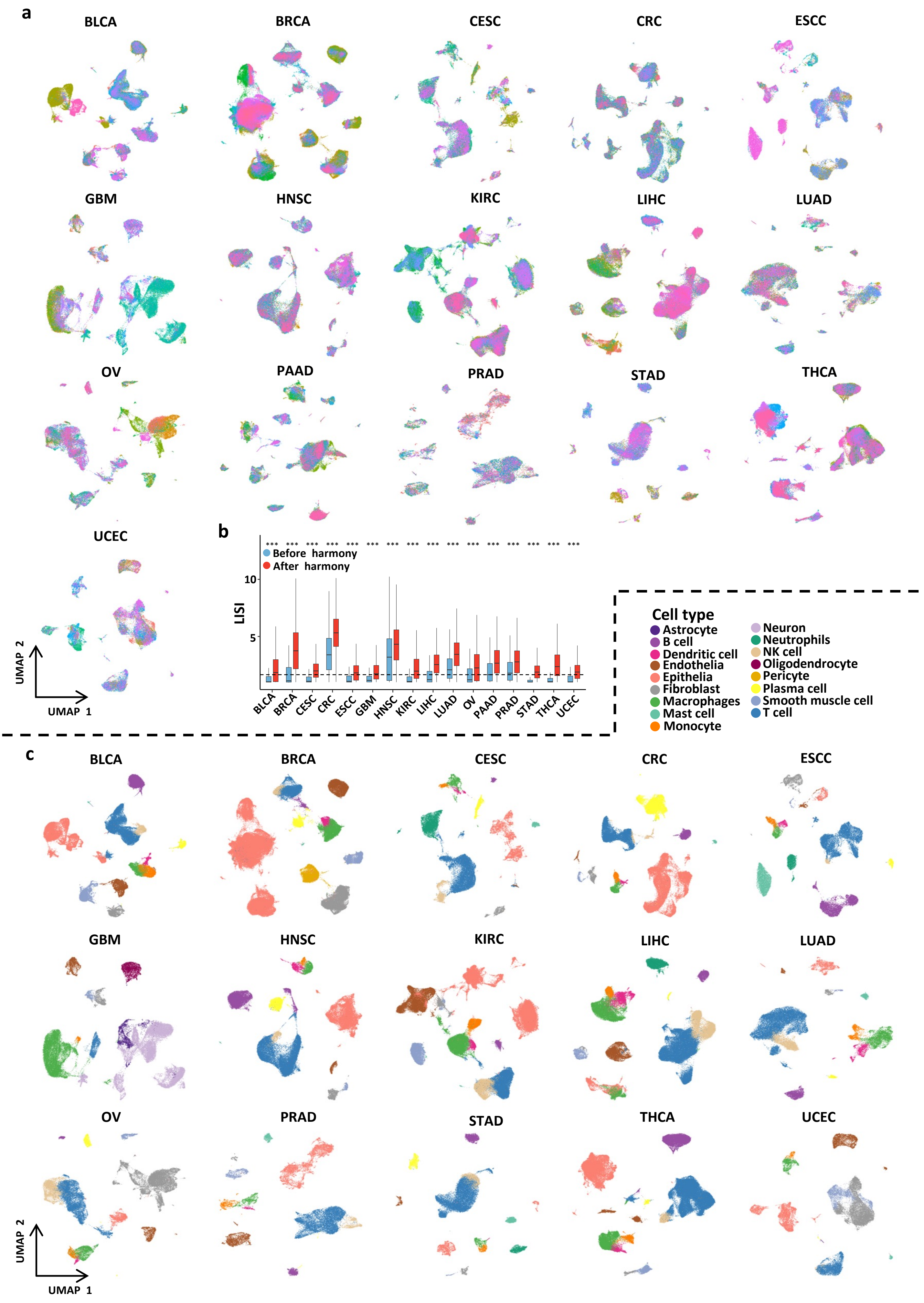
a
BLCA
BRCA
CESC
CRC
ESCC
GBM
HNSC
KIRC
LIHC
LUAD
OV
PAAD
PRAD
STAD
THCA
UCEC
UMAP 2
UMAP 1
b
Before harmony
After harmony
LISI
10
5
***
BLCA BRCA CESC CRC ESCC GBM HNSC KIRC LIHC LUAD OV PAAD PRAD STAD THCA UCEC
Cell type
Astrocyte
B cell
Dendritic cell
Endothelia
Epithelia
Fibroblast
Macrophages
Mast cell
Monocyte
Neuron
Neutrophils
NK cell
Oligodendrocyte
Pericyte
Plasma cell
Smooth muscle cell
T cell
c
BLCA
BRCA
CESC
CRC
ESCC
GBM
HNSC
KIRC
LIHC
LUAD
OV
PRAD
STAD
THCA
UCEC
UMAP 2
UMAP 1

Supplementary Fig. S1. scRNA-seq preprocessing and single-cell subtype definition, related to Fig. 1.

a UMAP plot illustrating the integration of samples across different cancer types, with colors representing distinct samples. **b** Boxplot displaying the batch effect levels assessed using the LISI index, where a mean value greater than 1 indicates minimal batch effects (*$P < 0.05$, **$P < 0.01$, ***$P < 0.001$). **c** UMAP plot demonstrating the major cell types identified in the example dataset across different cancer types, with colors representing distinct cell types.

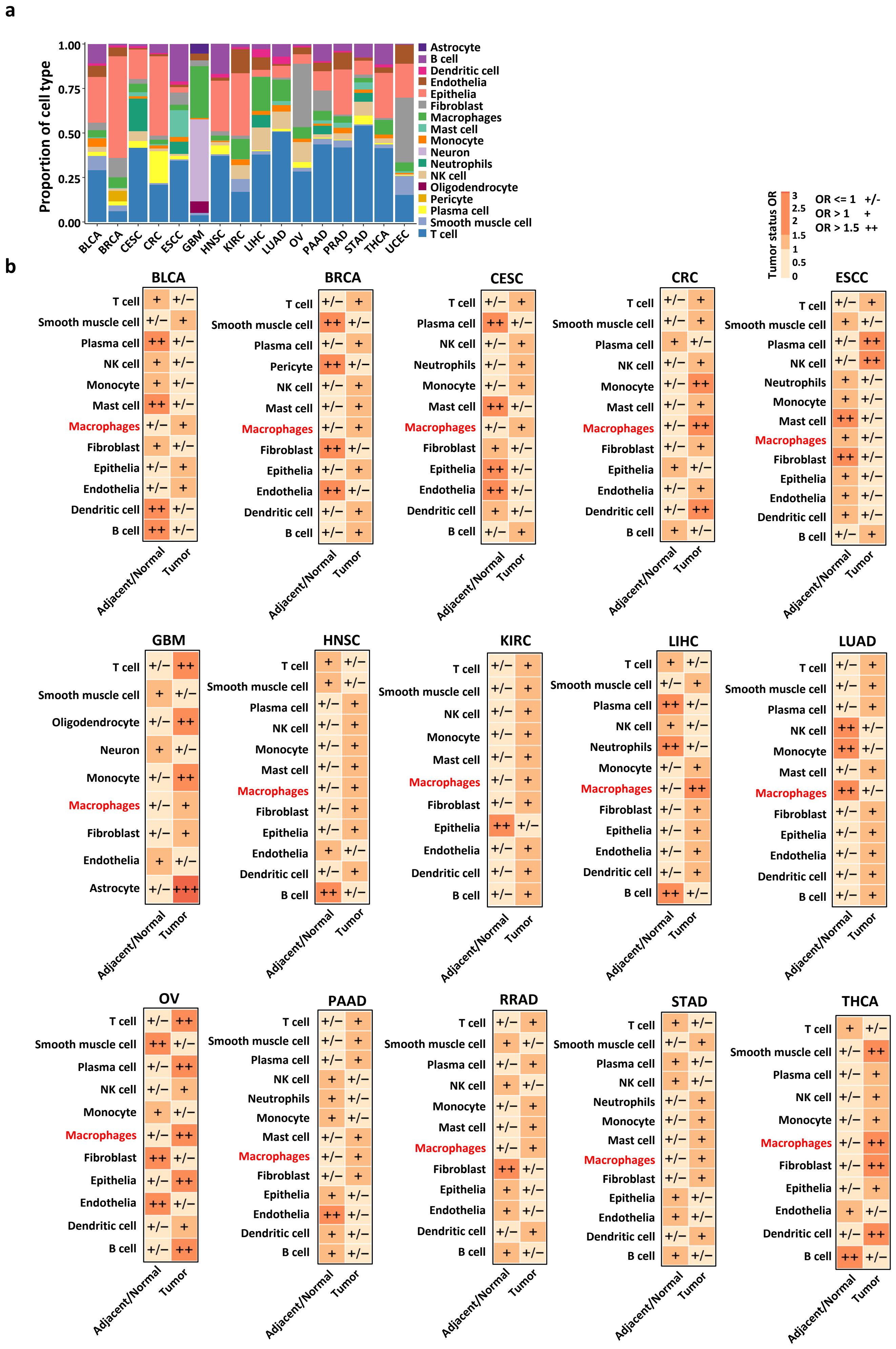

a
Proportion of cell type
1.00
0.75
0.50
0.25
0.00
BLCA
BRCA
CESC
CRC
ESCC
GBM
HNSC
KIRC
LIHC
LUAD
OV
PAAD
PRAD
STAD
THCA
UCEC
Astrocyte
B cell
Dendritic cell
Endothelia
Epithelia
Fibroblast
Macrophages
Mast cell
Monocyte
Neuron
Neutrophils
NK cell
Oligodendrocyte
Pericyte
Plasma cell
Smooth muscle cell
T cell
b
Tumor status OR
3
2.5
2
1.5
1
0.5
0
OR <= 1 +/-
OR > 1 +
OR > 1.5 ++
BLCA
BRCA
CESC
CRC
ESCC
GBM
HNSC
KIRC
LIHC
LUAD
OV
PAAD
RRAD
STAD
THCA
Adjacent/Normal
Tumor

Supplementary Fig. S2. Pan-cancer cell type proportions and preference analysis, related to Fig. 1.

a Bar plot illustrating the proportion of different cell types across various cancer types. **b** Heatmap showing the tissue preference of different cell types across various cancer types, where values greater than 1 indicate significant tissue preference**.**

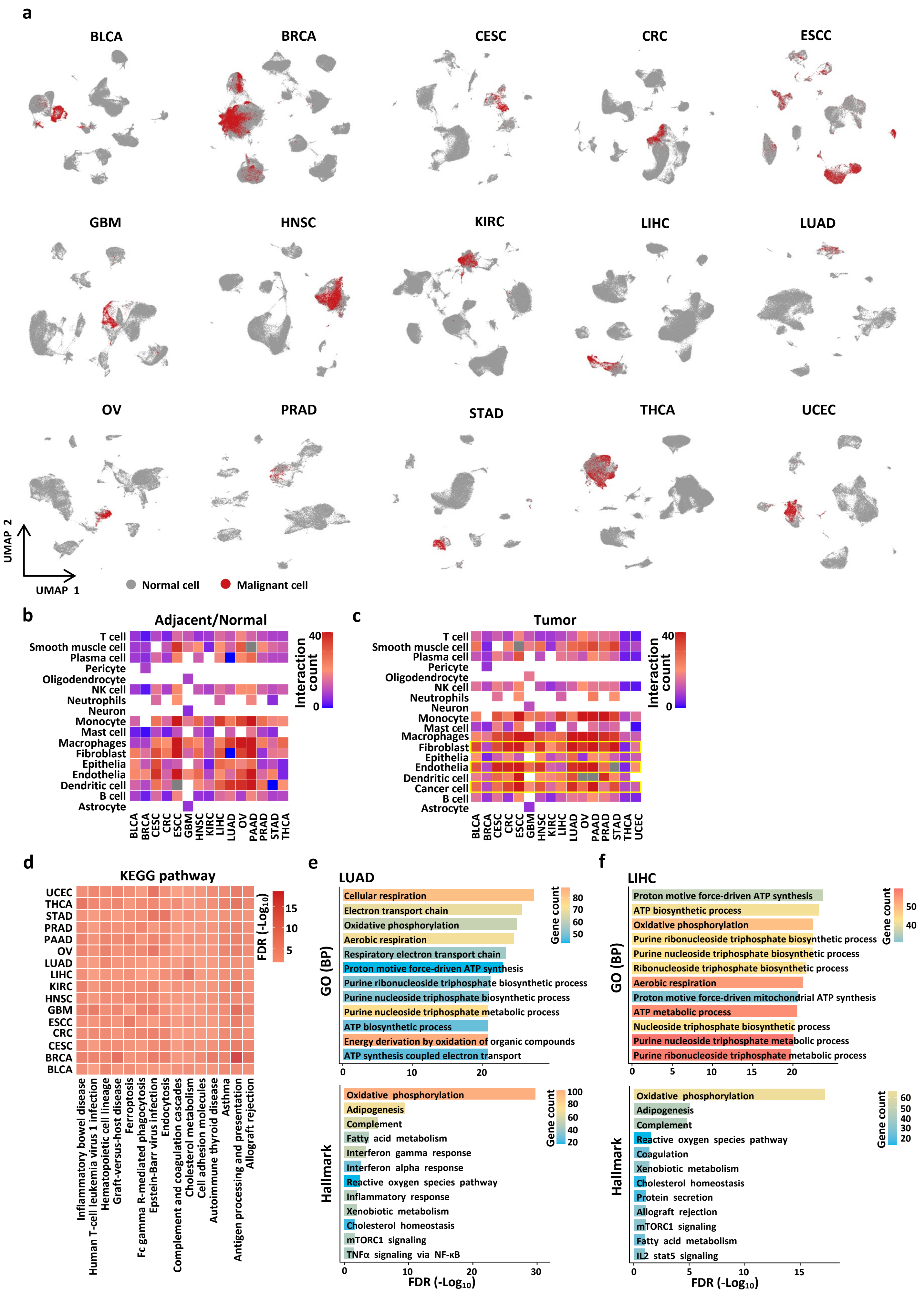
a
BLCA
BRCA
CESC
CRC
ESCC
GBM
HNSC
KIRC
LIHC
LUAD
OV
PRAD
STAD
THCA
UCEC
UMAP 2
UMAP 1
Normal cell
Malignant cell
b
Adjacent/Normal
T cell
Smooth muscle cell
Plasma cell
Pericyte
Oligodendrocyte
NK cell
Neutrophils
Neuron
Monocyte
Mast cell
Macrophages
Fibroblast
Epithelia
Endothelia
Dendritic cell
B cell
Astrocyte
BLCA BRCA CESC CRC ESCC GBM HNSC KIRC LIHC LUAD OV PAAD PRAD STAD THCA
Interaction count
40
0
c
Tumor
T cell
Smooth muscle cell
Plasma cell
Pericyte
Oligodendrocyte
NK cell
Neutrophils
Neuron
Monocyte
Mast cell
Macrophages
Fibroblast
Epithelia
Endothelia
Dendritic cell
Cancer cell
B cell
Astrocyte
BLCA BRCA CESC CRC ESCC GBM HNSC KIRC LIHC LUAD OV PAAD PRAD STAD THCA UCEC
Interaction count
40
0
d
KEGG pathway
UCEC
THCA
STAD
PRAD
PAAD
OV
LUAD
LIHC
KIRC
HNSC
GBM
ESCC
CRC
CESC
BRCA
BLCA
FDR (-Log10)
15
10
5
Inflammatory bowel disease
Human T-cell leukemia virus 1 infection
Hematopoietic cell lineage
Graft-versus-host disease
Ferroptosis
Fc gamma R-mediated phagocytosis
Epstein-Barr virus infection
Endocytosis
Complement and coagulation cascades
Cholesterol metabolism
Cell adhesion molecules
Autoimmune thyroid disease
Asthma
Antigen processing and presentation
Allograft rejection
e
LUAD
GO (BP)
Cellular respiration
Electron transport chain
Oxidative phosphorylation
Aerobic respiration
Respiratory electron transport chain
Proton motive force-driven ATP synthesis
Purine ribonucleoside triphosphate biosynthetic process
Purine nucleoside triphosphate biosynthetic process
Purine nucleoside triphosphate metabolic process
ATP biosynthetic process
Energy derivation by oxidation of organic compounds
ATP synthesis coupled electron transport
0 10 20
Gene count
80
70
60
50
Hallmark
Oxidative phosphorylation
Adipogenesis
Complement
Fatty acid metabolism
Interferon gamma response
Interferon alpha response
Reactive oxygen species pathway
Inflammatory response
Xenobiotic metabolism
Cholesterol homeostasis
mTORC1 signaling
TNFα signaling via NF-κB
0 10 20 30
Gene count
100
80
60
40
20
FDR (-Log10)
f
LIHC
GO (BP)
Proton motive force-driven ATP synthesis
ATP biosynthetic process
Oxidative phosphorylation
Purine ribonucleoside triphosphate biosynthetic process
Purine nucleoside triphosphate biosynthetic process
Ribonucleoside triphosphate biosynthetic process
Aerobic respiration
Proton motive force-driven mitochondrial ATP synthesis
ATP metabolic process
Nucleoside triphosphate biosynthetic process
Purine nucleoside triphosphate metabolic process
Purine ribonucleoside triphosphate metabolic process
0 5 10 15 20
Gene count
50
40
Hallmark
Oxidative phosphorylation
Adipogenesis
Complement
Reactive oxygen species pathway
Coagulation
Xenobiotic metabolism
Cholesterol homeostasis
Protein secretion
Allograft rejection
mTORC1 signaling
Fatty acid metabolism
IL2 stat5 signaling
0 5 10 15
Gene count
60
50
40
30
20
FDR (-Log10)

Supplementary Fig. S3. Malignant cell identification and macrophage signature analysis, related to Fig. 1.

a UMAP plot displaying malignant cells inferred using CopyKAT, where red represents cells with malignant features and gray represents cells without malignant tendencies. **b, c** Heatmap shows the interaction strength between macrophages and other microenvironmental cell types (y-axis) across different cancer types (x-axis) under adjacent or normal (b) and tumor tissue conditions (c), inferred using CellChat. **d** Enrichment analysis of macrophages across different cancer types using clusterProfiler, with pathways sourced from KEGG. **e, f** Separate enrichment analysis of macrophages in LUAD (e) and LIHC (f) using clusterProfiler, with pathways sourced from Gene Ontology (GO) and Hallmark.

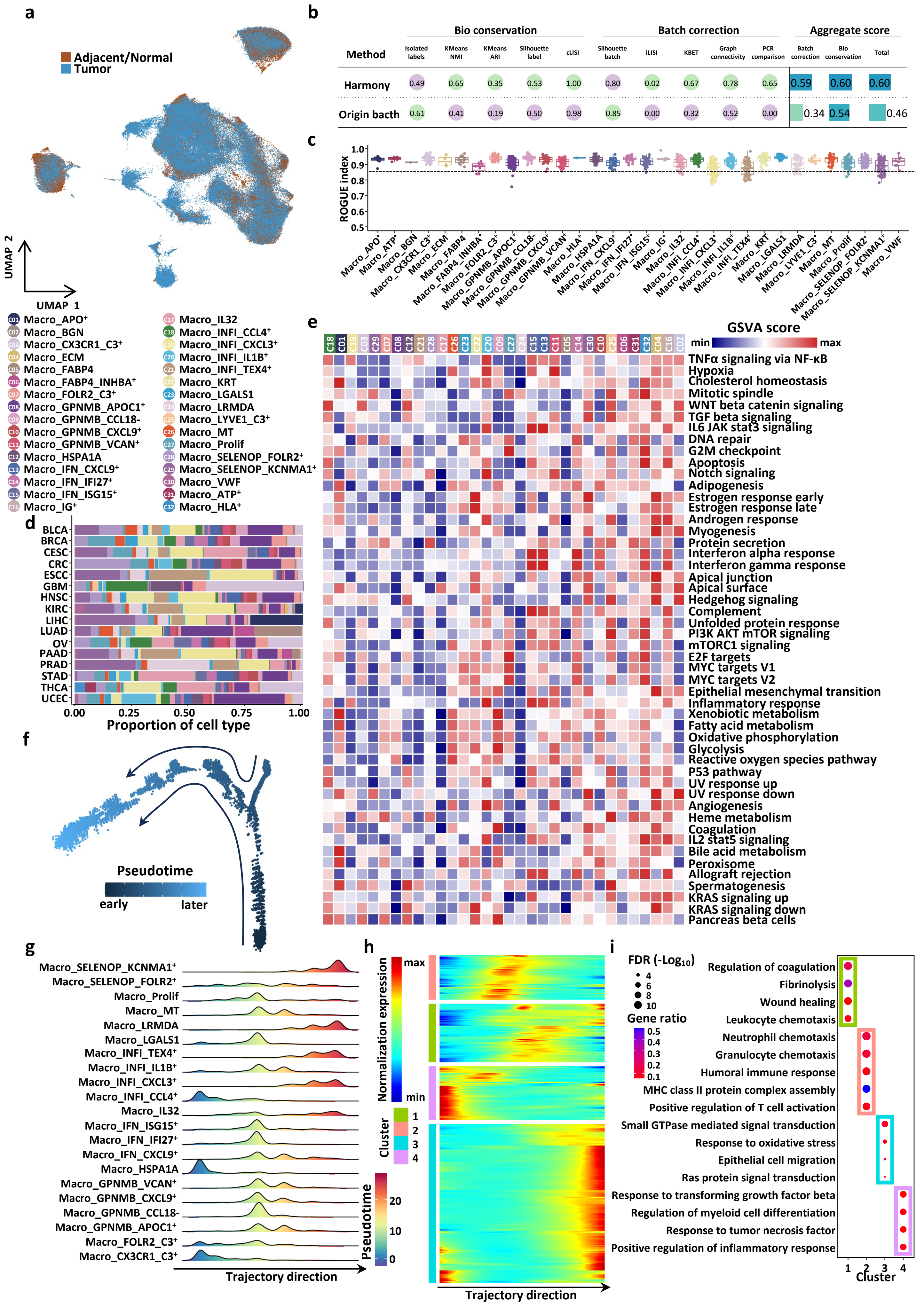
a
Adjacent/Normal
Tumor
UMAP 2
UMAP 1
C01 Macro_APO+
C02 Macro_BGN
C03 Macro_CX3CR1_C3+
C04 Macro_ECM
C05 Macro_FABP4
C06 Macro_FABP4_INHBA+
C07 Macro_FOLR2_C3+
C08 Macro_GPNMB_APOC1+
C09 Macro_GPNMB_CCL18-
C10 Macro_GPNMB_CXCL9+
C11 Macro_GPNMB_VCAN+
C12 Macro_HSPA1A
C13 Macro_IFN_CXCL9+
C14 Macro_IFN_IFI27+
C15 Macro_IFN_ISG15+
C16 Macro_IG+
C17 Macro_IL32
C18 Macro_INFI_CCL4+
C19 Macro_INFI_CXCL3+
C20 Macro_INFI_IL1B+
C21 Macro_INFI_TEX4+
C22 Macro_KRT
C23 Macro_LGALS1
C24 Macro_LRMDA
C25 Macro_LYVE1_C3+
C26 Macro_MT
C27 Macro_Prolif
C28 Macro_SELENOP_FOLR2+
C29 Macro_SELENOP_KCNMA1+
C30 Macro_VWF
C31 Macro_ATP+
C32 Macro_HLA+
b
Bio conservation
Batch correction
Aggregate score
Method
Isolated labels
KMeans NMI
KMeans ARI
Silhouette label
cLISI
Silhouette batch
iLISI
KBET
Graph connectivity
PCR comparison
Batch correction
Bio conservation
Total
Harmony 0.49 0.65 0.35 0.53 1.00 0.80 0.02 0.67 0.78 0.65 0.59 0.60 0.60
Origin bacth 0.61 0.41 0.19 0.50 0.98 0.85 0.00 0.32 0.52 0.00 0.34 0.54 0.46
c
ROGUE index
d
BLCA
BRCA
CESC
CRC
ESCC
GBM
HNSC
KIRC
LIHC
LUAD
OV
PAAD
PRAD
STAD
THCA
UCEC
Proportion of cell type
e
GSVA score
min
max
TNFα signaling via NF-κB
Hypoxia
Cholesterol homeostasis
Mitotic spindle
WNT beta catenin signaling
TGF beta signaling
IL6 JAK stat3 signaling
DNA repair
G2M checkpoint
Apoptosis
Notch signaling
Adipogenesis
Estrogen response early
Estrogen response late
Androgen response
Myogenesis
Protein secretion
Interferon alpha response
Interferon gamma response
Apical junction
Apical surface
Hedgehog signaling
Complement
Unfolded protein response
PI3K AKT mTOR signaling
mTORC1 signaling
E2F targets
MYC targets V1
MYC targets V2
Epithelial mesenchymal transition
Inflammatory response
Xenobiotic metabolism
Fatty acid metabolism
Oxidative phosphorylation
Glycolysis
Reactive oxygen species pathway
P53 pathway
UV response up
UV response down
Angiogenesis
Heme metabolism
Coagulation
IL2 stat5 signaling
Bile acid metabolism
Peroxisome
Allograft rejection
Spermatogenesis
KRAS signaling up
KRAS signaling down
Pancreas beta cells
f
Pseudotime
early
later
g
Trajectory direction
Pseudotime
h
Normalization expression
max
min
Cluster
1
2
3
4
Trajectory direction
i
FDR (-Log10)
Gene ratio
Regulation of coagulation
Fibrinolysis
Wound healing
Leukocyte chemotaxis
Neutrophil chemotaxis
Granulocyte chemotaxis
Humoral immune response
MHC class II protein complex assembly
Positive regulation of T cell activation
Small GTPase mediated signal transduction
Response to oxidative stress
Epithelial cell migration
Ras protein signal transduction
Response to transforming growth factor beta
Regulation of myeloid cell differentiation
Response to tumor necrosis factor
Positive regulation of inflammatory response
Cluster

Supplementary Fig. S4. Characterization of TAM features and biological functions at a pan-cancer level, related to Fig. 2.

a UMAP plot showing the mixing level of cells from tumor tissues and normal or adjacent tissues within the myeloid cell population. Blue represents cells from tumor tissues, and brown represents cells from adjacent normal or healthy tissues. **b** Use scIB framework to evaluate the effect of cross-tissue data integration before and after Harmony batch correction. **c** Boxplots showing cell purity for each TAM subset across samples by ROGUE index. **d** Bar plot showing the proportion of different TAM subtypes across various cancer types. **e** Heatmap showing the association between different TAM subtypes and Hallmark pathways inferred using the ssGSEA algorithm. **f** UMAP plot displaying the developmental trajectory of macrophages inferred using Monocle. **g** Ridge plot showing the abundance of pan-cancer subtypes at different time points along the developmental trajectory. **h** Heatmap showing gene expression changes along the pseudotime trajectory, with GO and ridge plots. **i** Bubble plot displaying the functional enrichment analysis results for different clusters along the developmental trajectory.

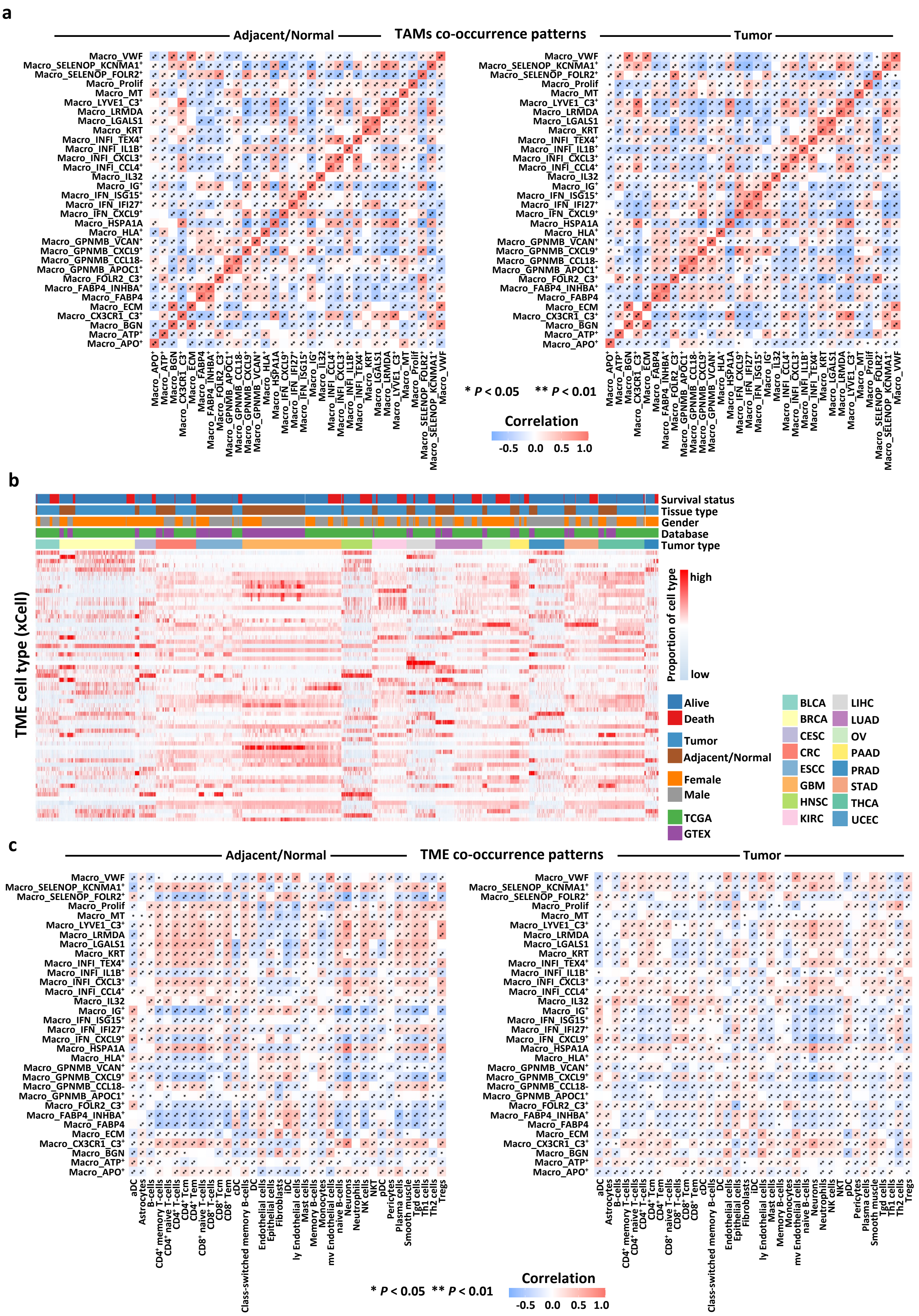

a
Adjacent/Normal
TAMs co-occurrence patterns
Tumor
Macro_VWF
Macro_SELENOP_KCNMA1+
Macro_SELENOP_FOLR2+
Macro_Prolif
Macro_MT
Macro_LYVE1_C3+
Macro_LRMDA
Macro_LGALS1
Macro_KRT
Macro_INFI_TEX4+
Macro_INFI_IL1B+
Macro_INFI_CXCL3+
Macro_INFI_CCL4+
Macro_IL32
Macro_IG+
Macro_IFN_ISG15+
Macro_IFN_IFI27+
Macro_IFN_CXCL9+
Macro_HSPA1A
Macro_HLA+
Macro_GPNMB_VCAN+
Macro_GPNMB_CXCL9+
Macro_GPNMB_CCL18-
Macro_GPNMB_APOC1+
Macro_FOLR2_C3+
Macro_FABP4_INHBA+
Macro_FABP4
Macro_ECM
Macro_CX3CR1_C3+
Macro_BGN
Macro_ATP+
Macro_APO+
* P < 0.05 ** P < 0.01
Correlation
-0.5 0.0 0.5 1.0
b
Survival status
Tissue type
Gender
Database
Tumor type
TME cell type (xCell)
Proportion of cell type
high
low
Alive
Death
Tumor
Adjacent/Normal
Female
Male
TCGA
GTEX
BLCA
BRCA
CESC
CRC
ESCC
GBM
HNSC
KIRC
LIHC
LUAD
OV
PAAD
PRAD
STAD
THCA
UCEC
c
Adjacent/Normal
TME co-occurrence patterns
Tumor
aDC
Astrocytes
B-cells
CD4+ memory T-cells
CD4+ naive T-cells
CD4+ T-cells
CD4+ Tcm
CD4+ Tem
CD8+ naive T-cells
CD8+ T-cells
CD8+ Tcm
CD8+ Tem
cDC
Class-switched memory B-cells
DC
Endothelial cells
Epithelial cells
Fibroblasts
iDC
ly Endothelial cells
Mast cells
Memory B-cells
Monocytes
mv Endothelial cells
naive B-cells
Neurons
Neutrophils
NK cells
NKT
pDC
Pericytes
Plasma cells
Smooth muscle
Tgd cells
Th1 cells
Th2 cells
Tregs
* P < 0.05 ** P < 0.01
Correlation
-0.5 0.0 0.5 1.0

Supplementary Fig. S5. Inference of cell type proportions in bulk RNA-seq using xCell, related to Fig. 3.

a Pearson correlation analysis of the co-occurrence patterns of different TAM subtypes in normal or adjacent tissues (left) and tumor tissues (right) (*$P < 0.05$, **$P < 0.01$). **b** Heatmap displaying the proportions of different TME components in TCGA and GTEx bulk RNA-seq samples inferred using the xCell algorithm. The left side represents all cell types included in xCell (Supplementary Table S9). **c** Pearson correlation analysis of the co-occurrence patterns between different hub TAM subtypes and TME components (inferred by Xcell) in normal or adjacent tissues (left) and tumor tissues (right) (*$P < 0.05$, **$P < 0.01$).

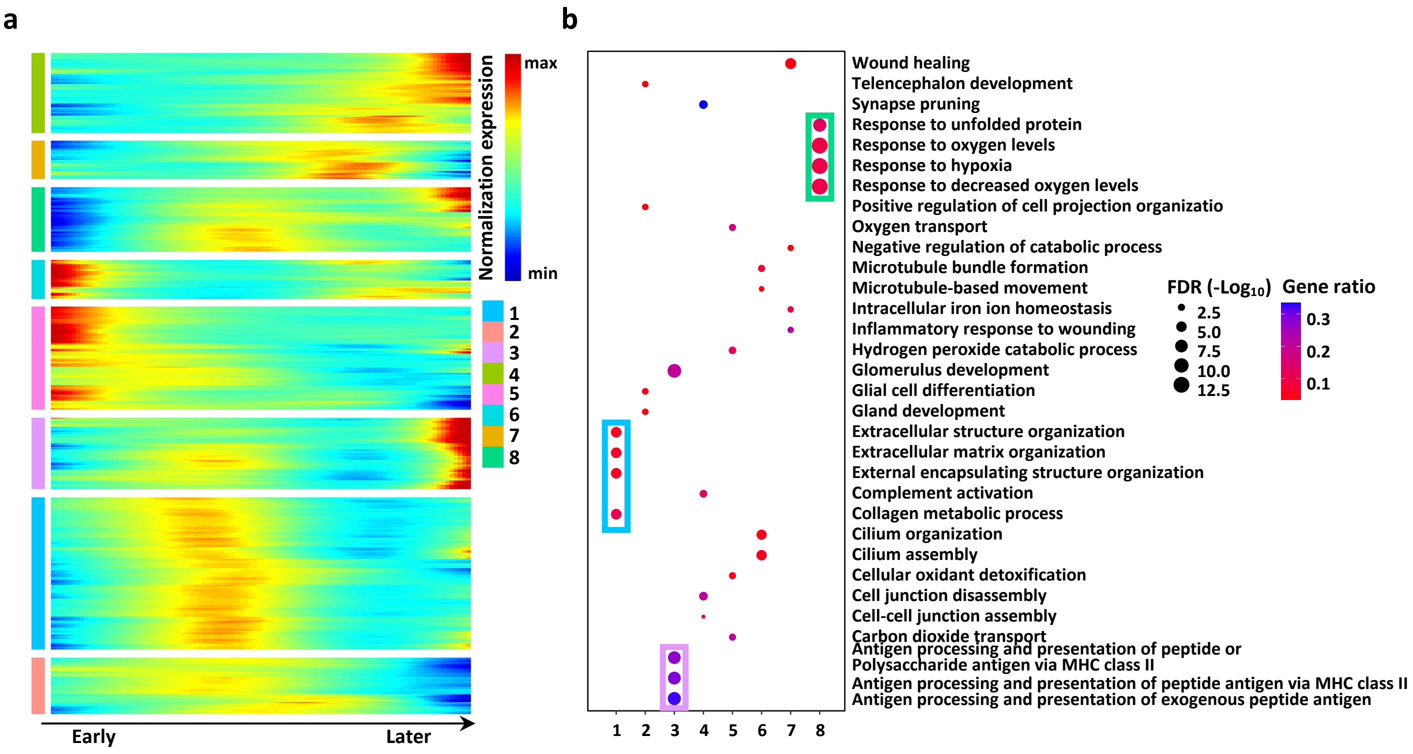

Supplementary Fig. S6. Developmental features of TAMs and activation of specific biological functions at the spatial level, related to Fig. 4.

a Heatmap showing gene expression across different clusters along the developmental trajectory axis (left to right: late development). **b** Bubble plot displaying GO enrichment analysis for different clusters along the developmental trajectory axis (clusters 1, 3 and 8 are primarily concentrated in late development).

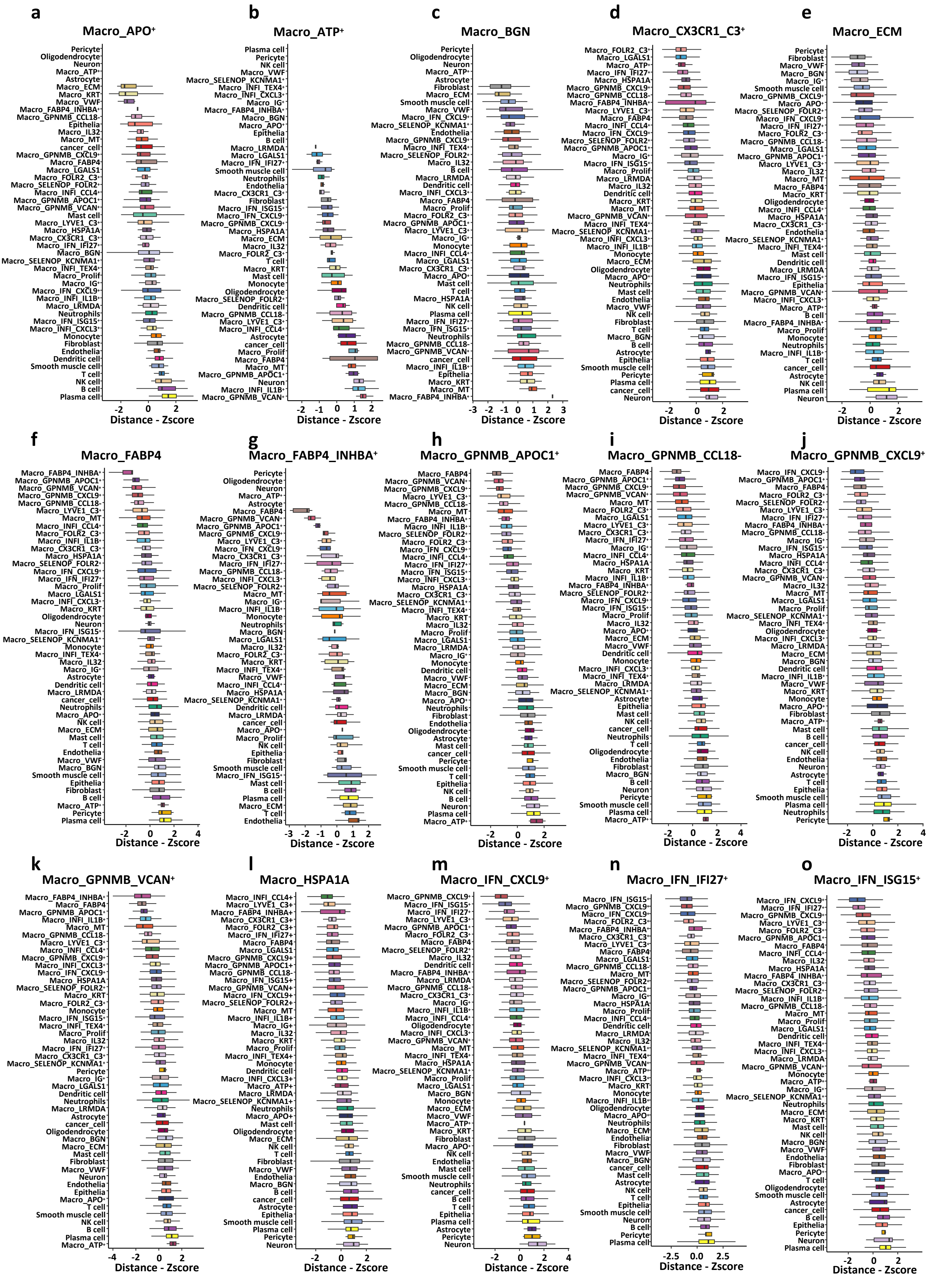
a
Macro_APO+
Pericyte
Oligodendrocyte
Neuron
Macro_ATP+
Astrocyte
Macro_ECM
Macro_KRT
Macro_VWF
Macro_FABP4_INHBA+
Macro_GPNMB_CCL18-
Epithelia
Macro_IL32
Macro_MT
cancer_cell
Macro_GPNMB_CXCL9+
Macro_FABP4
Macro_LGALS1
Macro_FOLR2_C3+
Macro_SELENOP_FOLR2+
Macro_INFI_CCL4+
Macro_GPNMB_APOC1+
Macro_GPNMB_VCAN+
Mast cell
Macro_LYVE1_C3+
Macro_HSPA1A
Macro_CX3CR1_C3+
Macro_IFN_IFI27+
Macro_BGN
Macro_SELENOP_KCNMA1+
Macro_INFI_TEX4+
Macro_Prolif
Macro_IG+
Macro_IFN_CXCL9+
Macro_INFI_IL1B+
Macro_LRMDA
Neutrophils
Macro_IFN_ISG15+
Macro_INFI_CXCL3+
Monocyte
Fibroblast
Endothelia
Dendritic cell
Smooth muscle cell
T cell
NK cell
B cell
Plasma cell
-2
0
2
Distance - Zscore
b
Macro_ATP+
Plasma cell
Pericyte
NK cell
Macro_VWF
Macro_SELENOP_KCNMA1+
Macro_INFI_TEX4+
Macro_INFI_CXCL3+
Macro_IG+
Macro_FABP4_INHBA+
Macro_BGN
Macro_APO+
Epithelia
B cell
Macro_LRMDA
Macro_LGALS1
Macro_IFN_IFI27+
Smooth muscle cell
Endothelia
Macro_CX3CR1_C3+
Fibroblast
Macro_IFN_ISG15+
Macro_IFN_CXCL9+
Macro_GPNMB_CXCL9+
Macro_HSPA1A
Macro_ECM
Macro_IL32
Macro_FOLR2_C3+
T cell
Macro_KRT
Mast cell
Monocyte
Oligodendrocyte
Macro_SELENOP_FOLR2+
Dendritic cell
Macro_GPNMB_CCL18-
Macro_LYVE1_C3+
Macro_INFI_CCL4+
Astrocyte
cancer_cell
Macro_Prolif
Macro_FABP4
Macro_MT
Macro_GPNMB_APOC1+
Neuron
Macro_INFI_IL1B+
Macro_GPNMB_VCAN+
-2
-1
0
1
2
Distance - Zscore
c
Macro_BGN
Pericyte
Oligodendrocyte
Neuron
Macro_ATP+
Astrocyte
Fibroblast
Macro_ECM
Smooth muscle cell
Macro_VWF
Macro_IFN_CXCL9+
Macro_SELENOP_KCNMA1+
Endothelia
Macro_GPNMB_CXCL9+
Macro_INFI_TEX4+
Macro_SELENOP_FOLR2+
Macro_IL32
B cell
Macro_LRMDA
Dendritic cell
Macro_INFI_CXCL3+
Macro_FABP4
Macro_Prolif
Macro_FOLR2_C3+
Macro_GPNMB_APOC1+
Macro_LYVE1_C3+
Macro_IG+
Monocyte
Macro_INFI_CCL4+
Macro_LGALS1
Macro_CX3CR1_C3+
Macro_APO+
Mast cell
T cell
Macro_HSPA1A
NK cell
Plasma cell
Macro_IFN_IFI27+
Macro_IFN_ISG15+
Neutrophils
Macro_GPNMB_CCL18-
Macro_GPNMB_VCAN+
cancer_cell
Macro_INFI_IL1B+
Epithelia
Macro_KRT
Macro_MT
Macro_FABP4_INHBA+
-3
-2
-1
0
1
2
3
Distance - Zscore
d
Macro_CX3CR1_C3+
Macro_FOLR2_C3+
Macro_LGALS1
Macro_ATP+
Macro_IFN_IFI27+
Macro_HSPA1A
Macro_GPNMB_CXCL9+
Macro_GPNMB_CCL18-
Macro_FABP4_INHBA+
Macro_LYVE1_C3+
Macro_FABP4
Macro_INFI_CCL4+
Macro_IFN_CXCL9+
Macro_SELENOP_FOLR2+
Macro_GPNMB_APOC1+
Macro_IG+
Macro_IFN_ISG15+
Macro_Prolif
Macro_LRMDA
Macro_IL32
Dendritic cell
Macro_KRT
Macro_MT
Macro_GPNMB_VCAN+
Macro_INFI_TEX4+
Macro_SELENOP_KCNMA1+
Macro_INFI_CXCL3+
Macro_INFI_IL1B+
Monocyte
Macro_ECM
Oligodendrocyte
Macro_APO+
Neutrophils
Mast cell
Endothelia
Macro_VWF
NK cell
Fibroblast
T cell
Macro_BGN
B cell
Astrocyte
Epithelia
Smooth muscle cell
Pericyte
Plasma cell
cancer_cell
Neuron
-2
0
2
Distance - Zscore
e
Macro_ECM
Pericyte
Fibroblast
Macro_VWF
Macro_BGN
Macro_IG+
Smooth muscle cell
Macro_GPNMB_CXCL9+
Macro_APO+
Macro_SELENOP_FOLR2+
Macro_IFN_CXCL9+
Macro_IFN_IFI27+
Macro_FOLR2_C3+
Macro_GPNMB_CCL18-
Macro_LGALS1
Macro_GPNMB_APOC1+
Macro_LYVE1_C3+
Macro_IL32
Macro_MT
Macro_FABP4
Macro_KRT
Oligodendrocyte
Macro_INFI_CCL4+
Macro_HSPA1A
Macro_CX3CR1_C3+
Endothelia
Macro_SELENOP_KCNMA1+
Macro_INFI_TEX4+
Mast cell
Dendritic cell
Macro_LRMDA
Macro_IFN_ISG15+
Epithelia
Macro_GPNMB_VCAN+
Macro_INFI_CXCL3+
Macro_ATP+
B cell
Macro_FABP4_INHBA+
Macro_Prolif
Monocyte
Neutrophils
Macro_INFI_IL1B+
T cell
cancer_cell
Astrocyte
NK cell
Plasma cell
Neuron
-2
0
2
Distance - Zscore
f
Macro_FABP4
Macro_FABP4_INHBA+
Macro_GPNMB_APOC1+
Macro_GPNMB_VCAN+
Macro_GPNMB_CXCL9+
Macro_GPNMB_CCL18-
Macro_LYVE1_C3+
Macro_MT
Macro_INFI_CCL4+
Macro_FOLR2_C3+
Macro_INFI_IL1B+
Macro_CX3CR1_C3+
Macro_HSPA1A
Macro_SELENOP_FOLR2+
Macro_IFN_CXCL9+
Macro_IFN_IFI27+
Macro_Prolif
Macro_LGALS1
Macro_INFI_CXCL3+
Macro_KRT
Oligodendrocyte
Neuron
Macro_IFN_ISG15+
Macro_SELENOP_KCNMA1+
Monocyte
Macro_INFI_TEX4+
Macro_IL32
Macro_IG+
Astrocyte
Dendritic cell
Macro_LRMDA
cancer_cell
Neutrophils
Macro_APO+
NK cell
Macro_ECM
Mast cell
T cell
Endothelia
Macro_VWF
Macro_BGN
Smooth muscle cell
Epithelia
Fibroblast
B cell
Macro_ATP+
Pericyte
Plasma cell
-2
0
2
4
Distance - Zscore
g
Macro_FABP4_INHBA+
Pericyte
Oligodendrocyte
Neuron
Macro_ATP+
Astrocyte
Macro_FABP4
Macro_GPNMB_VCAN+
Macro_GPNMB_APOC1+
Macro_GPNMB_CXCL9+
Macro_LYVE1_C3+
Macro_IFN_CXCL9+
Macro_CX3CR1_C3+
Macro_IFN_IFI27+
Macro_GPNMB_CCL18-
Macro_INFI_CXCL3+
Macro_SELENOP_FOLR2+
Macro_MT
Macro_IG+
Macro_INFI_IL1B+
Monocyte
Neutrophils
Macro_BGN
Macro_LGALS1
Macro_IL32
Macro_FOLR2_C3+
Macro_KRT
Macro_INFI_TEX4+
Macro_VWF
Macro_INFI_CCL4+
Macro_HSPA1A
Macro_SELENOP_KCNMA1+
Dendritic cell
Macro_LRMDA
cancer_cell
Macro_APO+
Macro_Prolif
NK cell
Epithelia
Fibroblast
Smooth muscle cell
Macro_IFN_ISG15+
Mast cell
B cell
Plasma cell
Macro_ECM
T cell
Endothelia
-3
-2
-1
0
1
2
Distance - Zscore
h
Macro_GPNMB_APOC1+
Macro_FABP4
Macro_GPNMB_VCAN+
Macro_GPNMB_CXCL9+
Macro_LYVE1_C3+
Macro_GPNMB_CCL18-
Macro_MT
Macro_FABP4_INHBA+
Macro_INFI_IL1B+
Macro_SELENOP_FOLR2+
Macro_FOLR2_C3+
Macro_IFN_CXCL9+
Macro_INFI_CCL4+
Macro_IFN_IFI27+
Macro_IFN_ISG15+
Macro_INFI_CXCL3+
Macro_HSPA1A
Macro_CX3CR1_C3+
Macro_SELENOP_KCNMA1+
Macro_INFI_TEX4+
Macro_KRT
Macro_IL32
Macro_Prolif
Macro_LGALS1
Macro_LRMDA
Macro_IG+
Monocyte
Dendritic cell
Macro_VWF
Macro_ECM
Macro_BGN
Macro_APO+
Neutrophils
Fibroblast
Endothelia
Oligodendrocyte
Astrocyte
Mast cell
cancer_cell
Pericyte
Smooth muscle cell
T cell
Epithelia
NK cell
B cell
Neuron
Plasma cell
Macro_ATP+
-2
0
2
Distance - Zscore
i
Macro_GPNMB_CCL18-
Macro_FABP4
Macro_GPNMB_APOC1+
Macro_GPNMB_CXCL9+
Macro_GPNMB_VCAN+
Macro_MT
Macro_FOLR2_C3+
Macro_LGALS1
Macro_LYVE1_C3+
Macro_CX3CR1_C3+
Macro_IFN_IFI27+
Macro_IG+
Macro_INFI_CCL4+
Macro_HSPA1A
Macro_KRT
Macro_INFI_IL1B+
Macro_FABP4_INHBA+
Macro_SELENOP_FOLR2+
Macro_IFN_CXCL9+
Macro_IFN_ISG15+
Macro_Prolif
Macro_IL32
Macro_APO+
Macro_ECM
Macro_VWF
Dendritic cell
Monocyte
Macro_INFI_CXCL3+
Macro_INFI_TEX4+
Macro_LRMDA
Macro_SELENOP_KCNMA1+
Astrocyte
Epithelia
Mast cell
NK cell
cancer_cell
Neutrophils
T cell
Oligodendrocyte
Endothelia
Fibroblast
Macro_BGN
B cell
Neuron
Pericyte
Smooth muscle cell
Plasma cell
Macro_ATP+
-2
0
2
4
Distance - Zscore
j
Macro_GPNMB_CXCL9+
Macro_IFN_CXCL9+
Macro_GPNMB_APOC1+
Macro_FABP4
Macro_FOLR2_C3+
Macro_SELENOP_FOLR2+
Macro_LYVE1_C3+
Macro_IFN_IFI27+
Macro_FABP4_INHBA+
Macro_GPNMB_CCL18-
Macro_IG+
Macro_IFN_ISG15+
Macro_HSPA1A
Macro_INFI_CCL4+
Macro_CX3CR1_C3+
Macro_GPNMB_VCAN+
Macro_IL32
Macro_MT
Macro_LGALS1
Macro_Prolif
Macro_SELENOP_KCNMA1+
Macro_INFI_TEX4+
Oligodendrocyte
Macro_INFI_CXCL3+
Macro_LRMDA
Macro_ECM
Macro_BGN
Dendritic cell
Macro_INFI_IL1B+
Macro_VWF
Macro_KRT
Monocyte
Macro_APO+
Fibroblast
Macro_ATP+
Mast cell
B cell
cancer_cell
NK cell
Endothelia
Neuron
Astrocyte
T cell
Epithelia
Smooth muscle cell
Plasma cell
Neutrophils
Pericyte
-2
0
2
4
Distance - Zscore
k
Macro_GPNMB_VCAN+
Macro_FABP4_INHBA+
Macro_FABP4
Macro_GPNMB_APOC1+
Macro_INFI_IL1B+
Macro_MT
Macro_GPNMB_CCL18-
Macro_LYVE1_C3+
Macro_INFI_CCL4+
Macro_GPNMB_CXCL9+
Macro_INFI_CXCL3+
Macro_IFN_CXCL9+
Macro_HSPA1A
Macro_SELENOP_FOLR2+
Macro_KRT
Macro_FOLR2_C3+
Monocyte
Macro_IFN_ISG15+
Macro_INFI_TEX4+
Macro_Prolif
Macro_IL32
Macro_IFN_IFI27+
Macro_CX3CR1_C3+
Macro_SELENOP_KCNMA1+
Pericyte
Macro_IG+
Macro_LGALS1
Dendritic cell
Neutrophils
Macro_LRMDA
Astrocyte
cancer_cell
Oligodendrocyte
Macro_BGN
Macro_ECM
Mast cell
Fibroblast
Macro_VWF
Neuron
Endothelia
Epithelia
Macro_APO+
T cell
Smooth muscle cell
NK cell
B cell
Plasma cell
Macro_ATP+
-4
-2
0
2
Distance - Zscore
l
Macro_HSPA1A
Macro_INFI_CCL4+
Macro_LYVE1_C3+
Macro_FABP4_INHBA+
Macro_CX3CR1_C3+
Macro_FOLR2_C3+
Macro_IFN_IFI27+
Macro_FABP4
Macro_LGALS1
Macro_GPNMB_CXCL9+
Macro_GPNMB_APOC1+
Macro_GPNMB_CCL18-
Macro_IFN_ISG15+
Macro_GPNMB_VCAN+
Macro_IFN_CXCL9+
Macro_SELENOP_FOLR2+
Macro_MT
Macro_INFI_IL1B+
Macro_IG+
Macro_IL32
Macro_KRT
Macro_Prolif
Macro_INFI_TEX4+
Monocyte
Dendritic cell
Macro_INFI_CXCL3+
Macro_ATP+
Macro_LRMDA
Macro_SELENOP_KCNMA1+
Neutrophils
Macro_APO+
Mast cell
Oligodendrocyte
Macro_ECM
NK cell
T cell
Fibroblast
Macro_VWF
Endothelia
Macro_BGN
B cell
cancer_cell
Astrocyte
Epithelia
Smooth muscle cell
Plasma cell
Pericyte
Neuron
-2
0
2
Distance - Zscore
m
Macro_IFN_CXCL9+
Macro_GPNMB_CXCL9+
Macro_IFN_ISG15+
Macro_IFN_IFI27+
Macro_LYVE1_C3+
Macro_GPNMB_APOC1+
Macro_FOLR2_C3+
Macro_FABP4
Macro_SELENOP_FOLR2+
Macro_IL32
Dendritic cell
Macro_FABP4_INHBA+
Macro_LRMDA
Macro_GPNMB_CCL18-
Macro_CX3CR1_C3+
Macro_IG+
Macro_INFI_IL1B+
Macro_INFI_CCL4+
Oligodendrocyte
Macro_INFI_CXCL3+
Macro_GPNMB_VCAN+
Macro_MT
Macro_INFI_TEX4+
Macro_HSPA1A
Macro_SELENOP_KCNMA1+
Macro_Prolif
Macro_LGALS1
Macro_BGN
Monocyte
Macro_ECM
Macro_VWF
Macro_ATP+
Macro_KRT
Fibroblast
Macro_APO+
NK cell
Endothelia
Mast cell
Smooth muscle cell
Neutrophils
cancer_cell
B cell
T cell
Epithelia
Plasma cell
Astrocyte
Pericyte
Neuron
-2
0
2
4
Distance - Zscore
n
Macro_IFN_IFI27+
Macro_IFN_ISG15+
Macro_GPNMB_CXCL9+
Macro_IFN_CXCL9+
Macro_FOLR2_C3+
Macro_FABP4_INHBA+
Macro_CX3CR1_C3+
Macro_LYVE1_C3+
Macro_FABP4
Macro_LGALS1
Macro_GPNMB_CCL18-
Macro_MT
Macro_SELENOP_FOLR2+
Macro_GPNMB_APOC1+
Macro_IG+
Macro_HSPA1A
Macro_Prolif
Macro_INFI_CCL4+
Dendritic cell
Macro_LRMDA
Macro_IL32
Macro_SELENOP_KCNMA1+
Macro_INFI_TEX4+
Macro_GPNMB_VCAN+
Macro_ATP+
Macro_INFI_CXCL3+
Macro_KRT
Monocyte
Macro_INFI_IL1B+
Oligodendrocyte
Macro_APO+
Neutrophils
Macro_ECM
Endothelia
Fibroblast
Macro_VWF
Macro_BGN
cancer_cell
Mast cell
Astrocyte
NK cell
T cell
Epithelia
Smooth muscle cell
Neuron
B cell
Pericyte
Plasma cell
-2.5
0.0
2.5
Distance - Zscore
o
Macro_IFN_ISG15+
Macro_IFN_CXCL9+
Macro_IFN_IFI27+
Macro_GPNMB_CXCL9+
Macro_LYVE1_C3+
Macro_FOLR2_C3+
Macro_GPNMB_APOC1+
Macro_FABP4
Macro_INFI_CCL4+
Macro_IL32
Macro_HSPA1A
Macro_FABP4_INHBA+
Macro_CX3CR1_C3+
Macro_SELENOP_FOLR2+
Macro_INFI_IL1B+
Macro_GPNMB_CCL18-
Macro_MT
Macro_Prolif
Macro_LGALS1
Dendritic cell
Macro_INFI_TEX4+
Macro_INFI_CXCL3+
Macro_LRMDA
Macro_GPNMB_VCAN+
Monocyte
Macro_ATP+
Macro_IG+
Macro_SELENOP_KCNMA1+
Neutrophils
Macro_ECM
Macro_KRT
Mast cell
NK cell
Macro_BGN
Macro_VWF
Endothelia
Fibroblast
Macro_APO+
T cell
Oligodendrocyte
Smooth muscle cell
Astrocyte
cancer_cell
B cell
Epithelia
Pericyte
Neuron
Plasma cell
-2
0
2
Distance - Zscore

Supplementary Fig. S7. Spatial relationships between TAM subtypes and other cell types at a pan-cancer level, related to Fig. 4.

a-o Boxplots showing the average spatial distances between different TAM subtypes and other TME components, with spatial distances sorted in descending order based on mean distance.

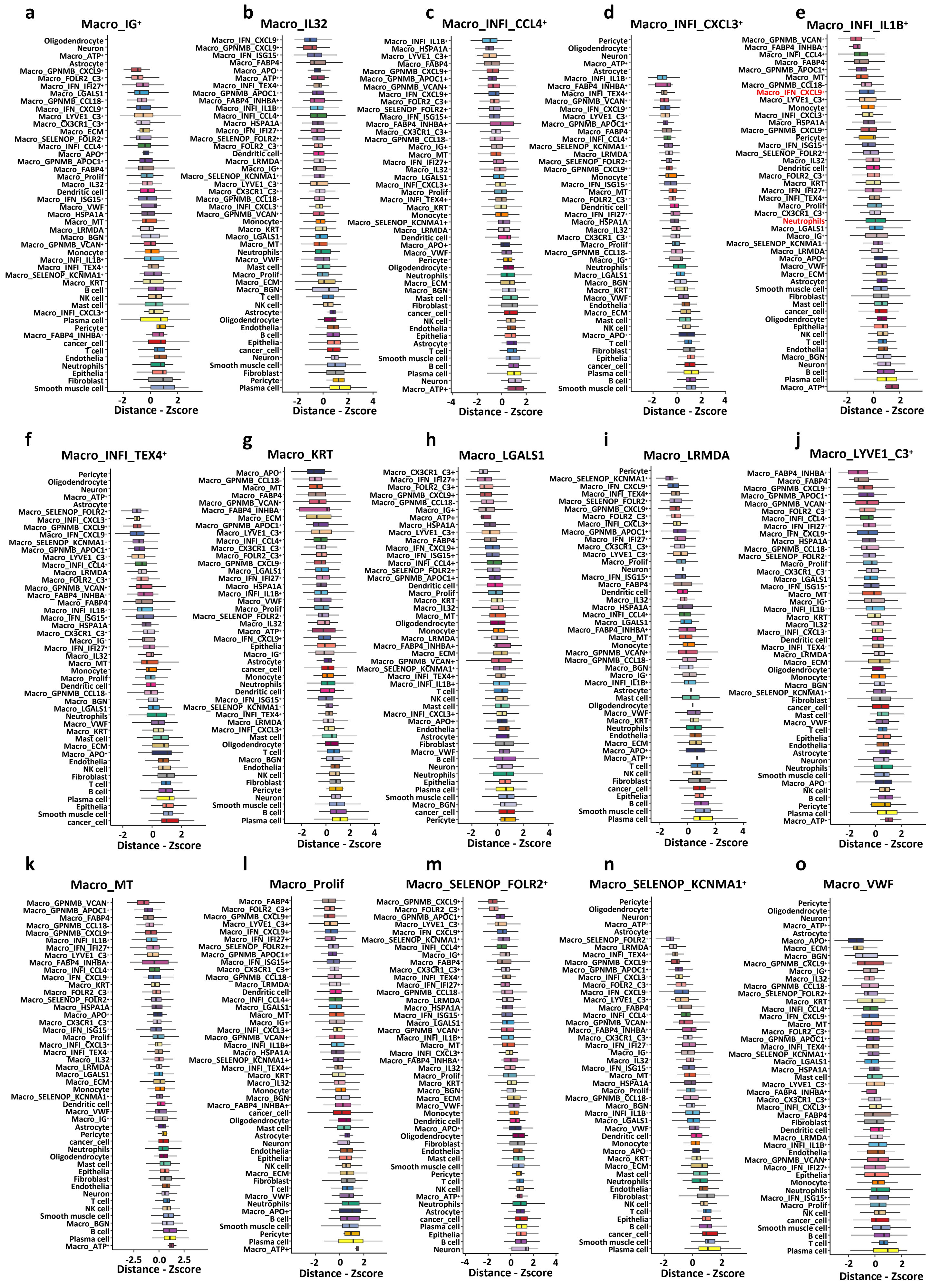

a
Macro_IG⁺
b
Macro_IL32
c
Macro_INFI_CCL4⁺
d
Macro_INFI_CXCL3⁺
e
Macro_INFI_IL1B⁺
f
Macro_INFI_TEX4⁺
g
Macro_KRT
h
Macro_LGALS1
i
Macro_LRMDA
j
Macro_LYVE1_C3⁺
k
Macro_MT
l
Macro_Prolif
m
Macro_SELENOP_FOLR2⁺
n
Macro_SELENOP_KCNMA1⁺
o
Macro_VWF
Distance - Zscore

Supplementary Fig. S8. Spatial relationships between TAM subtypes and other cell types at a pan-cancer level, related to Fig. 4.

a-o Boxplots showing the average spatial distances between different TAM subtypes and other TME components, with spatial distances sorted in descending order based on mean distance.

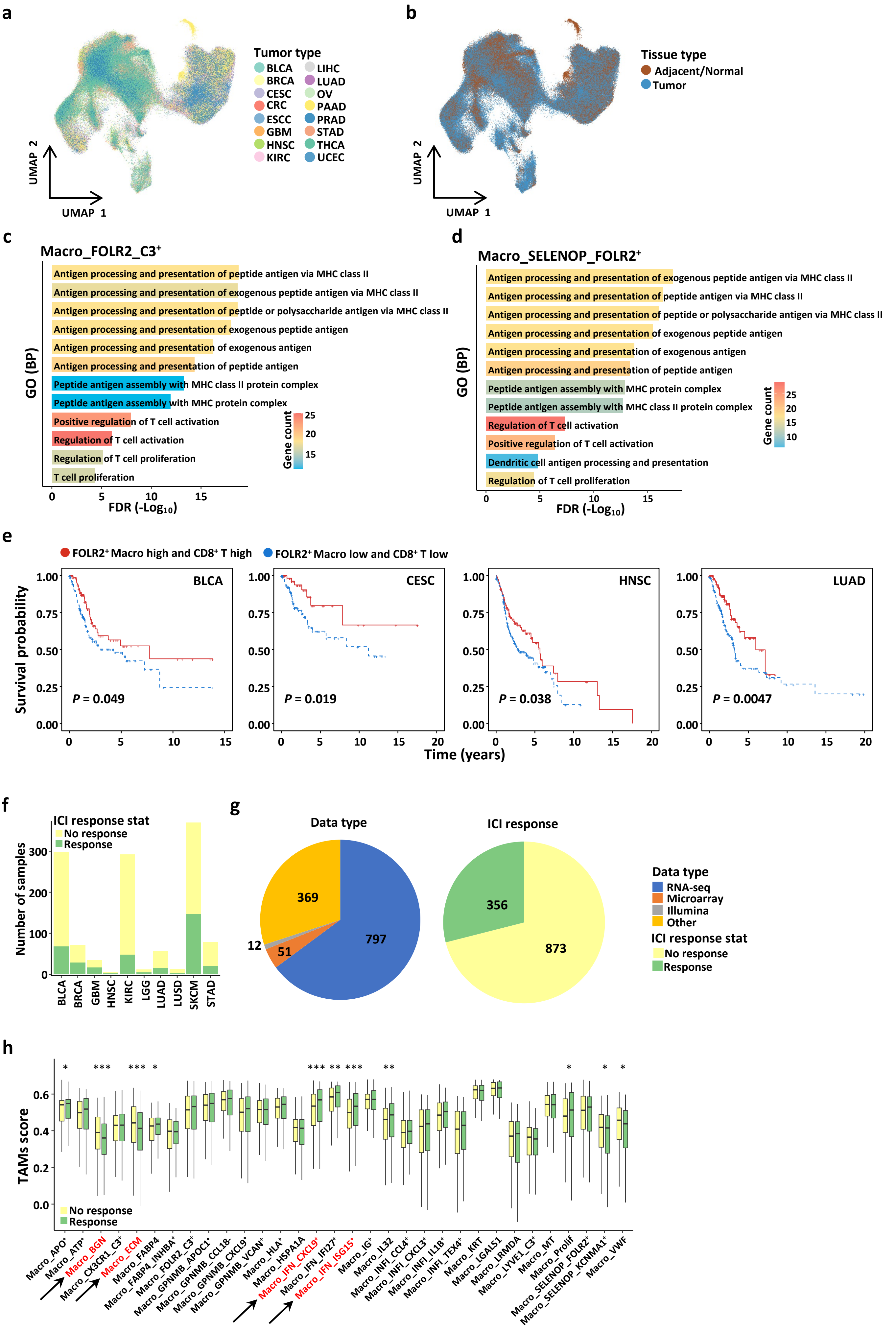
a
Tumor type
BLCA
BRCA
CESC
CRC
ESCC
GBM
HNSC
KIRC
LIHC
LUAD
OV
PAAD
PRAD
STAD
THCA
UCEC
UMAP 1
UMAP 2
b
Tissue type
Adjacent/Normal
Tumor
c
Macro_FOLR2_C3+
GO (BP)
Antigen processing and presentation of peptide antigen via MHC class II
Antigen processing and presentation of exogenous peptide antigen via MHC class II
Antigen processing and presentation of peptide or polysaccharide antigen via MHC class II
Antigen processing and presentation of exogenous peptide antigen
Antigen processing and presentation of exogenous antigen
Antigen processing and presentation of peptide antigen
Peptide antigen assembly with MHC class II protein complex
Peptide antigen assembly with MHC protein complex
Positive regulation of T cell activation
Regulation of T cell activation
Regulation of T cell proliferation
T cell proliferation
Gene count
FDR (-Log10)
d
Macro_SELENOP_FOLR2+
GO (BP)
Antigen processing and presentation of exogenous peptide antigen via MHC class II
Antigen processing and presentation of peptide antigen via MHC class II
Antigen processing and presentation of peptide or polysaccharide antigen via MHC class II
Antigen processing and presentation of exogenous peptide antigen
Antigen processing and presentation of exogenous antigen
Antigen processing and presentation of peptide antigen
Peptide antigen assembly with MHC protein complex
Peptide antigen assembly with MHC class II protein complex
Regulation of T cell activation
Positive regulation of T cell activation
Dendritic cell antigen processing and presentation
Regulation of T cell proliferation
Gene count
FDR (-Log10)
e
FOLR2+ Macro high and CD8+ T high
FOLR2+ Macro low and CD8+ T low
Survival probability
BLCA P = 0.049
CESC P = 0.019
HNSC P = 0.038
LUAD P = 0.0047
Time (years)
f
ICI response stat
No response
Response
Number of samples
BLCA
BRCA
GBM
HNSC
KIRC
LGG
LUAD
LUSD
SKCM
STAD
g
Data type
ICI response
369
797
51
12
356
873
Data type
RNA-seq
Microarray
Illumina
Other
ICI response stat
No response
Response
h
TAMs score
No response
Response
Macro_APO+
Macro_ATP+
Macro_BGN
Macro_CX3CR1_C3+
Macro_ECM
Macro_FABP4
Macro_FABP4_INHBA+
Macro_FOLR2_C3+
Macro_GPNMB_APOC1+
Macro_GPNMB_CCL18-
Macro_GPNMB_CXCL9+
Macro_GPNMB_VCAN+
Macro_HLA+
Macro_HSPA1A
Macro_IFN_CXCL9+
Macro_IFN_IFI27+
Macro_IFN_ISG15+
Macro_IG+
Macro_IL32
Macro_INFI_CCL4+
Macro_INFI_CXCL3+
Macro_INFI_IL1B+
Macro_INFI_TEX4+
Macro_KRT
Macro_LGALS1
Macro_LRMDA
Macro_LYVE1_C3+
Macro_MT
Macro_Prolif
Macro_SELENOP_FOLR2+
Macro_SELENOP_KCNMA1+
Macro_VWF

Supplementary Fig. S9. Potential interactions between TAMs and CD8$^+$ T cells and association with immunotherapy, related to Fig. 5.

a UMAP plot showing the mixing level of different cancer types within T cells. **b** UMAP plot showing the mixing of tumor tissues and normal or adjacent tissues within T cells. **c, d** Bar plots displaying the functional enrichment analysis results for Macro_FOLR2_C3$^+$ and Macro_SELENOP_FOLR2$^+$. **e** Kaplan-Meier curves showing the prognostic value of high abundance of FLOR3$^+$ Macro and CD8$^+$ T cells in immunotherapy cohorts, with *P*-values calculated using the log-rank test. **f** Bar plot displaying the number of patients receiving ICI treatment. **g** Left: Pie chart showing the data sources of patients receiving ICI treatment; Right: Pie chart showing the number of responders and non-responders to ICI treatment, with yellow representing non-responders and green representing responders. **h** Boxplots showing the abundance of different TAM subtypes in immunotherapy cohorts, with yellow representing non-responders and green representing responders (*$P < 0.05$, **$P < 0.01$, ***$P < 0.001$).

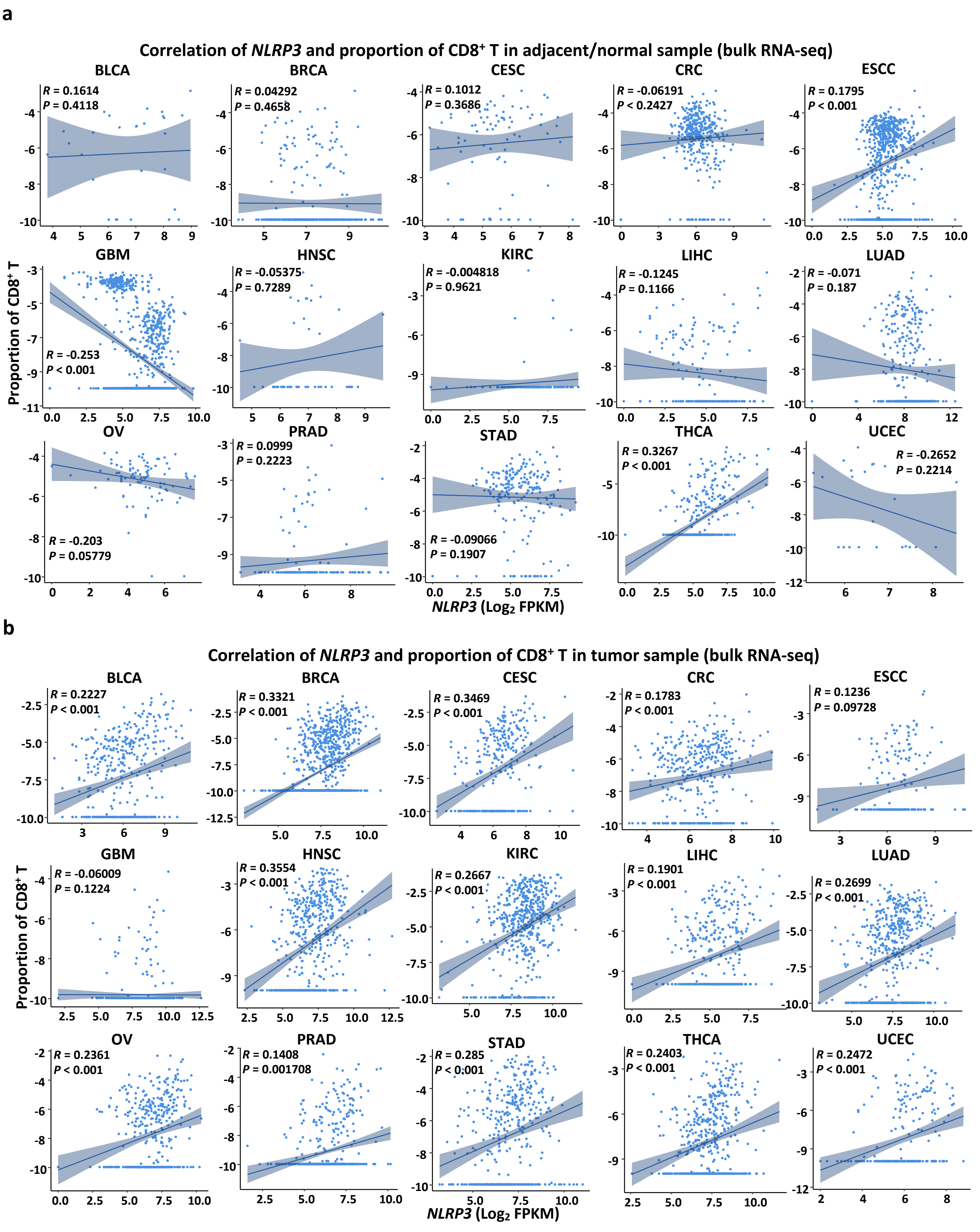

Supplementary Fig. S10. Correlation between inflammasome (*NLRP3*) and CD8$^+$ T cell abundance, related to Fig. 5.

a Correlation between CD8$^+$ T cell abundance and *NLRP3* expression in normal or adjacent tissues. **b** Correlation between CD8$^+$ T cell abundance and *NLRP3* expression in tumor tissues.

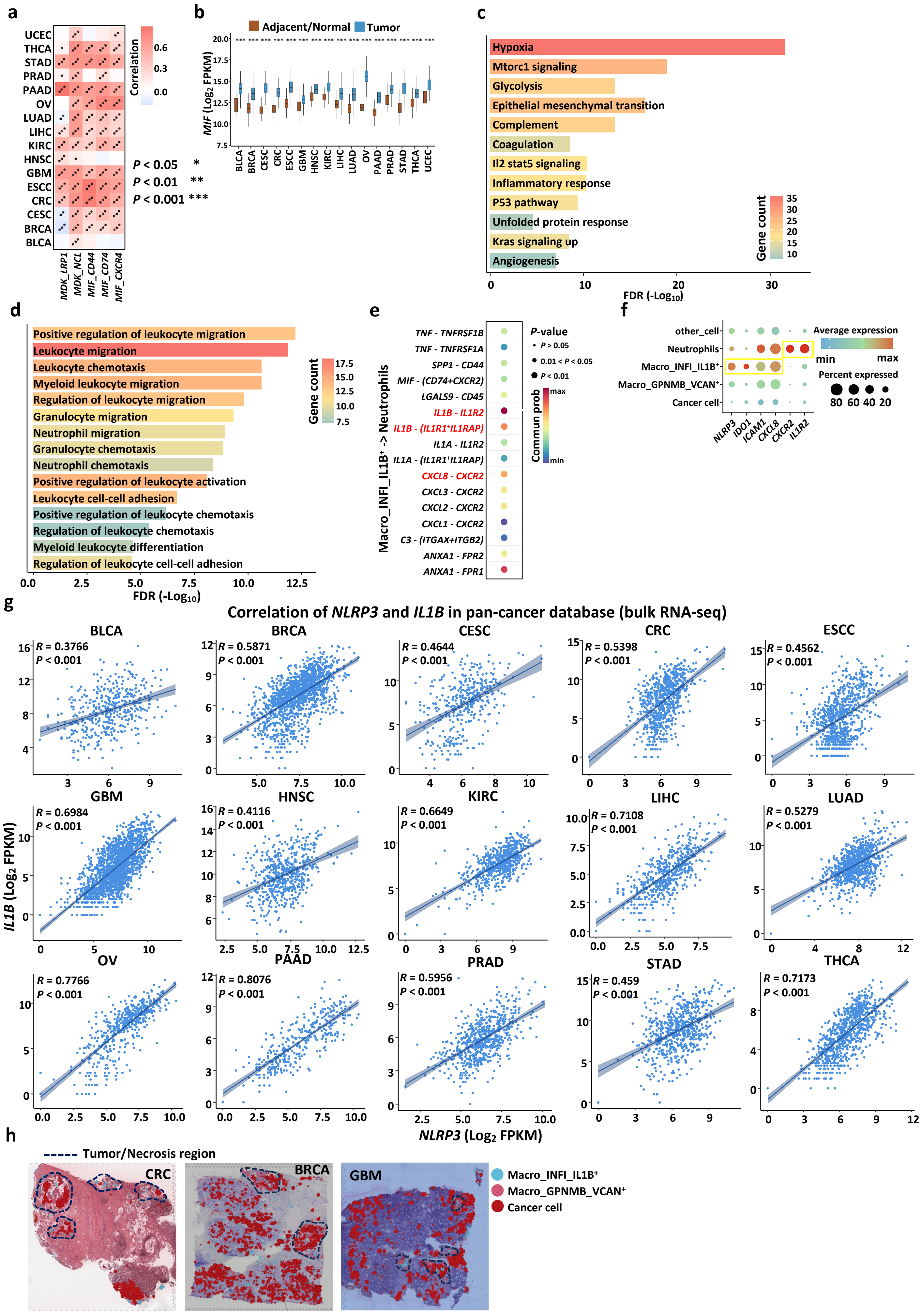
a
b
Adjacent/Normal
Tumor
c
Hypoxia
Mtorc1 signaling
Glycolysis
Epithelial mesenchymal transition
Complement
Coagulation
Il2 stat5 signaling
Inflammatory response
P53 pathway
Unfolded protein response
Kras signaling up
Angiogenesis
Gene count
FDR (-Log10)
d
Positive regulation of leukocyte migration
Leukocyte migration
Leukocyte chemotaxis
Myeloid leukocyte migration
Regulation of leukocyte migration
Granulocyte migration
Neutrophil migration
Granulocyte chemotaxis
Neutrophil chemotaxis
Positive regulation of leukocyte activation
Leukocyte cell-cell adhesion
Positive regulation of leukocyte chemotaxis
Regulation of leukocyte chemotaxis
Myeloid leukocyte differentiation
Regulation of leukocyte cell-cell adhesion
Gene count
FDR (-Log10)
e
Macro_INFI_IL1B+ -> Neutrophils
P-value
Commun prob
f
Average expression
Percent expressed
g
Correlation of NLRP3 and IL1B in pan-cancer database (bulk RNA-seq)
IL1B (Log2 FPKM)
NLRP3 (Log2 FPKM)
h
Tumor/Necrosis region
CRC
BRCA
GBM
Macro_INFI_IL1B+
Macro_GPNMB_VCAN+
Cancer cell

Supplementary Fig. S11. Interaction features between TAMs localized in the tumor core and cancer cells, related to Fig. 6.

a Heatmap showing the correlation between the expression of *MDK* and *MIF* ligands and their corresponding receptor genes (*$P < 0.05$, **$P < 0.01$, ***$P < 0.001$). **b** Boxplot showing the differential expression of *MIF* at a pan-cancer level (*$P < 0.05$, **$P < 0.01$, ***$P < 0.001$). **c** Hallmark enrichment analysis results for signature genes of Macro_GPNMB_VCAN$^+$. **d** GO enrichment analysis results for signature genes of Macro_INFI_IL1B$^+$. **e** Bubble plot showing the interaction ligands and receptors between Macro_INFI_IL1B$^+$ and neutrophils. **f** Bubble plot displaying the expression of key signature genes (*NLRP3*, *IDO1*, and *ICAM1*) and ligand-receptor pairs (*CXCL8*, *CXCR2*, and *IL1R2*) in Macro_INFI_IL1B$^+$ and neutrophils. **g** Correlation between *NLRP3* and *IL1B* gene expression at a pan-cancer level. **h** Annotation of necrotic regions on spatial slices, with red dashed lines marking the regions, the purple dashed area represents the tumor necrosis region.

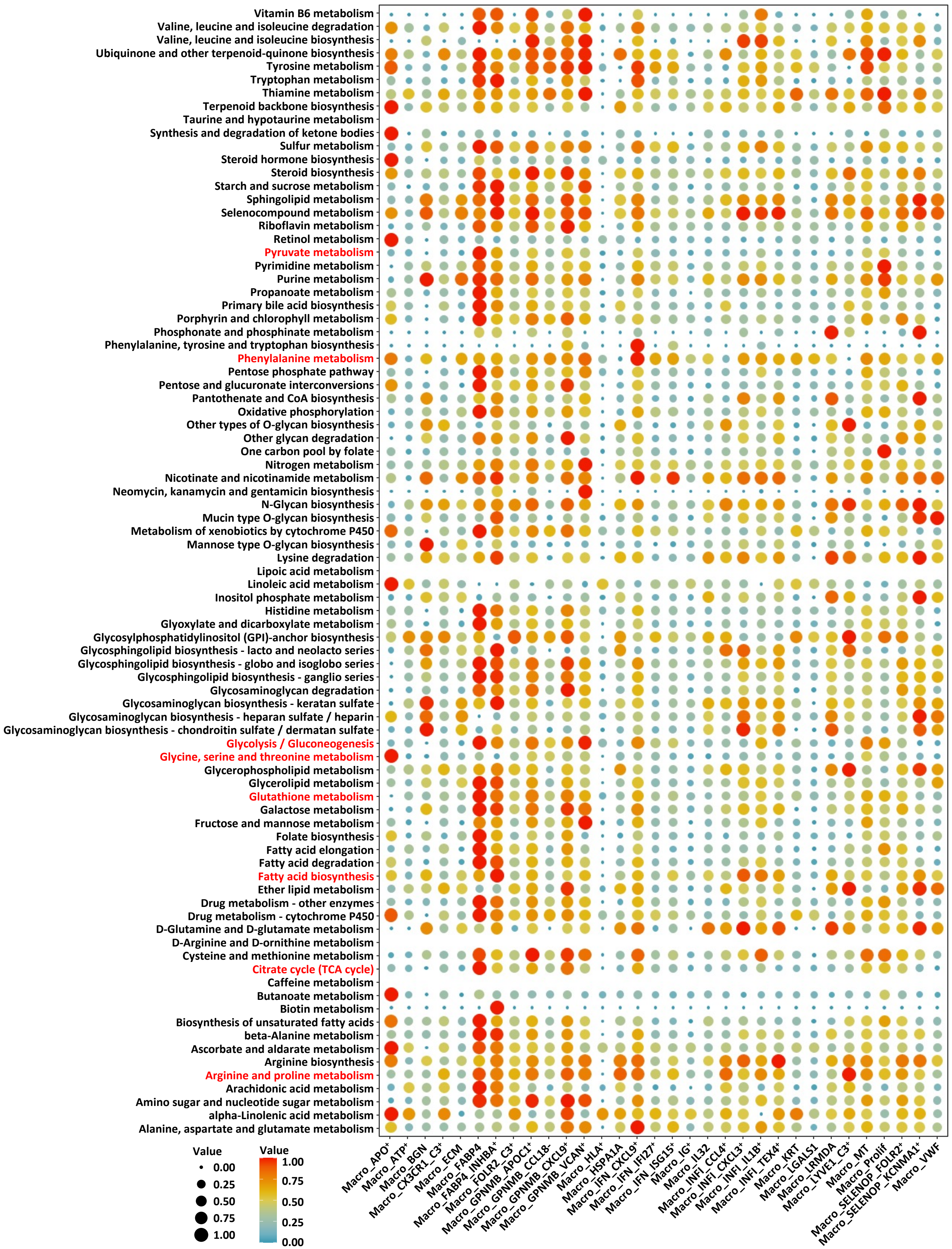

Supplementary Fig. S12. Involvement of TAMs in metabolic pathway activity, related to Fig. 6. Bubble plot displaying the metabolic pathway activity of different TAM subtypes analyzed using scMetabolism.

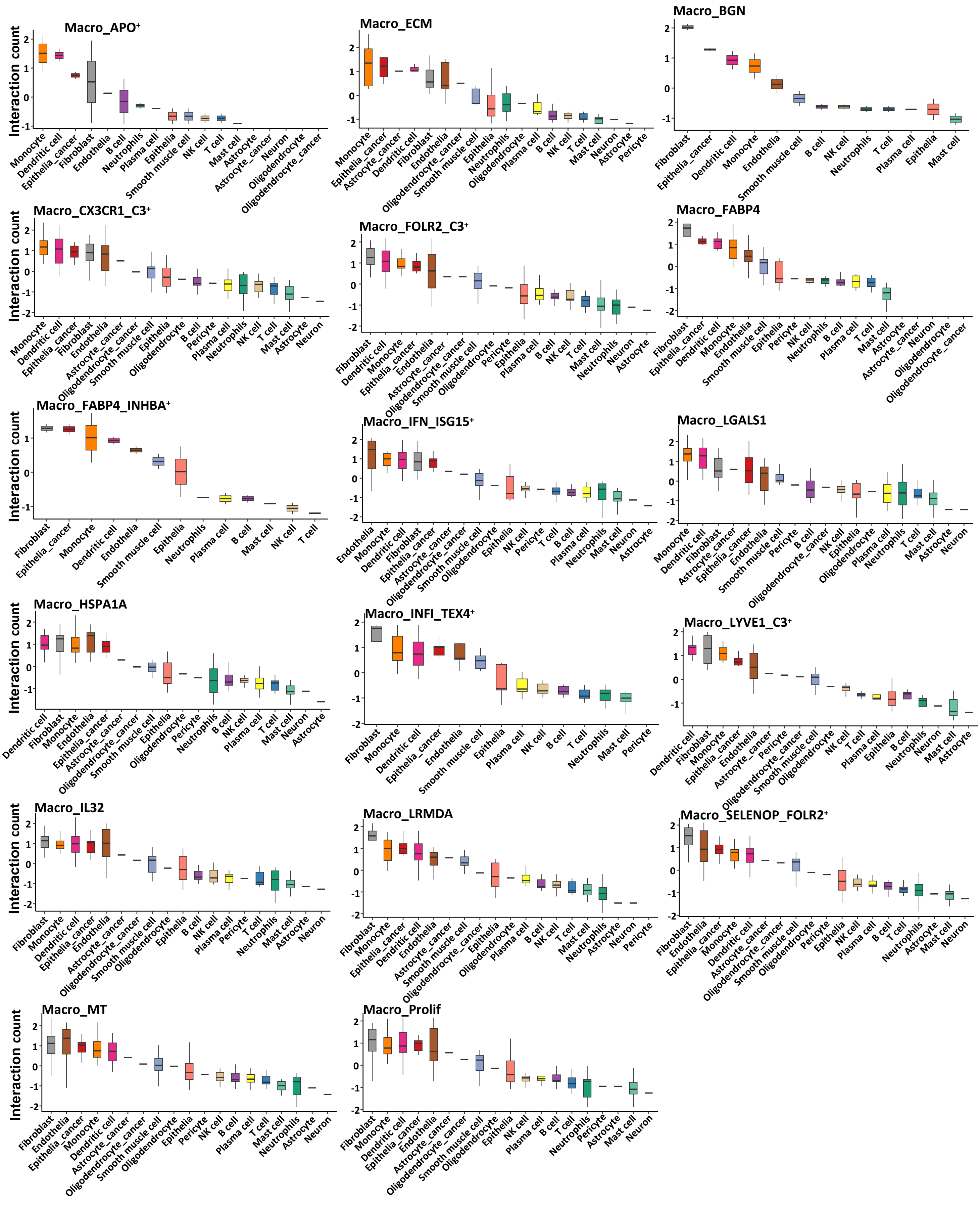

Supplementary Fig. S13. Interaction strength between TAM subtypes and TME components, related to Fig. 7.

Boxplot showing the interaction strength between different TAM subtypes and other TME components inferred using CellChat.

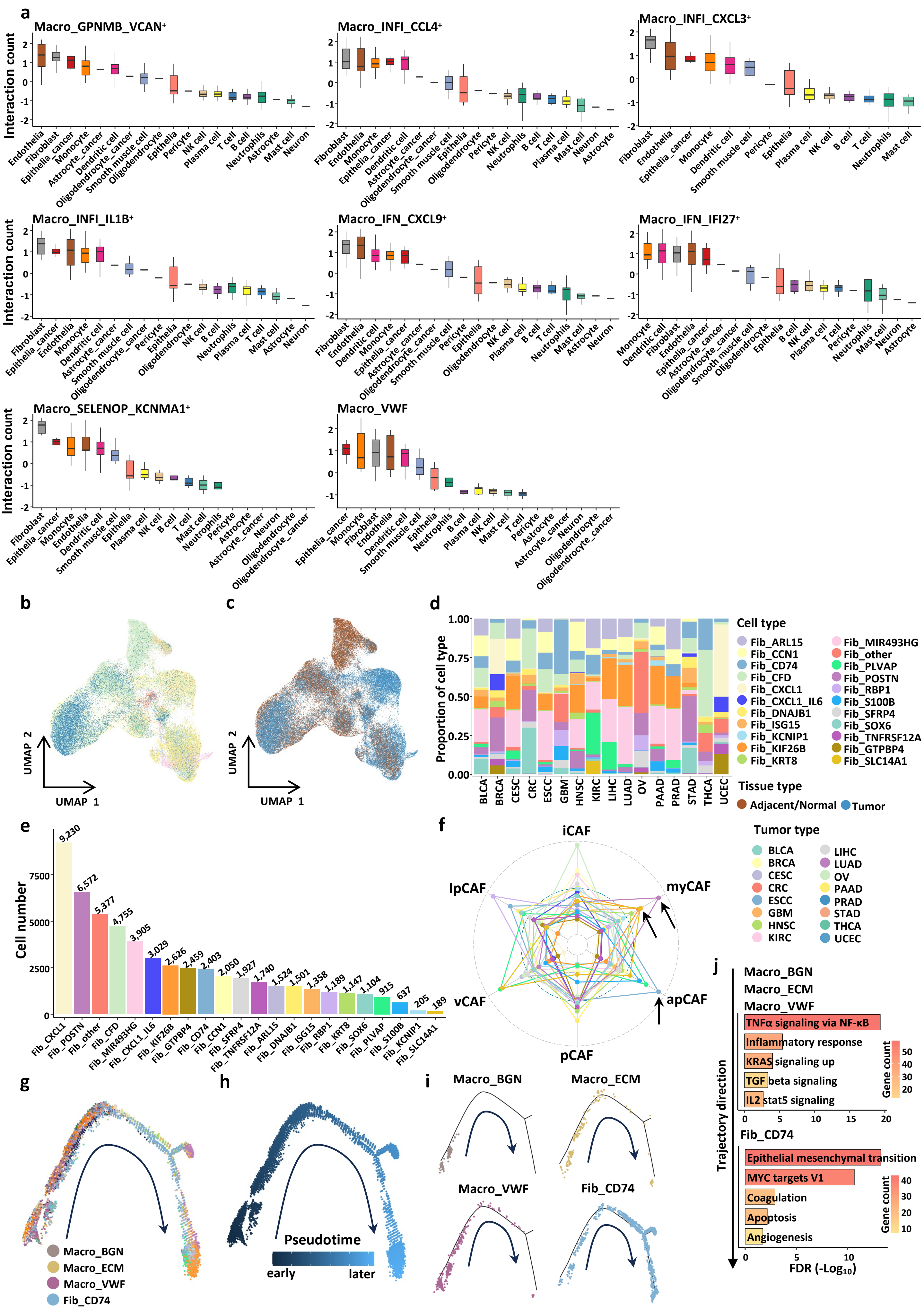
a
Macro_GPNMB_VCAN+
Macro_INFI_CCL4+
Macro_INFI_CXCL3+
Macro_INFI_IL1B+
Macro_IFN_CXCL9+
Macro_IFN_IFI27+
Macro_SELENOP_KCNMA1+
Macro_VWF
Interaction count
b
c
d
UMAP 1
UMAP 2
Proportion of cell type
Cell type
Fib_ARL15
Fib_CCN1
Fib_CD74
Fib_CFD
Fib_CXCL1
Fib_CXCL1_IL6
Fib_DNAJB1
Fib_ISG15
Fib_KCNIP1
Fib_KIF26B
Fib_KRT8
Fib_MIR493HG
Fib_other
Fib_PLVAP
Fib_POSTN
Fib_RBP1
Fib_S100B
Fib_SFRP4
Fib_SOX6
Fib_TNFRSF12A
Fib_GTPBP4
Fib_SLC14A1
Tissue type
Adjacent/Normal
Tumor
BLCA BRCA CESC CRC ESCC GBM HNSC KIRC LIHC LUAD OV PAAD PRAD STAD THCA UCEC
e
Cell number
9,230 6,572 5,377 4,755 3,905 3,029 2,626 2,459 2,403 2,050 1,927 1,740 1,524 1,501 1,358 1,189 1,147 1,104 915 637 205 189
f
iCAF
myCAF
apCAF
pCAF
vCAF
lpCAF
Tumor type
g
h
i
j
Macro_BGN
Macro_ECM
Macro_VWF
Fib_CD74
Pseudotime
early
later
Trajectory direction
TNFα signaling via NF-κB
Inflammatory response
KRAS signaling up
TGF beta signaling
IL2 stat5 signaling
Epithelial mesenchymal transition
MYC targets V1
Coagulation
Apoptosis
Angiogenesis
Gene count
FDR (-Log10)

Supplementary Fig. S14. Characterization and developmental states of CAFs, related to Fig. 7.

a Boxplot showing the interaction strength between different TAM subtypes and other TME components inferred using CellChat. **b** UMAP plot showing the mixing level of different cancer types within fibroblasts. **c** UMAP plot showing the mixing of tumor tissues and normal or adjacent tissues within fibroblasts. **d** Bar plot displaying the proportion of different fibroblast subtypes across various cancer types. **e** Bar plot showing the number of different fibroblast subtypes across various cancer types. **f** Radar plot showing the similarity between fibroblast subtypes defined in this study and the six traditionally defined subtypes. **g, h** Distribution of different cell types (left) and developmental direction (right) along the trajectory inferred using Monocle2. **i** Distribution of Macro_BGN, Macro_ECM, Macro_VWF, and Fib_CD74 along the developmental trajectory. **j** Enrichment analysis results of signature genes during the development of Macro_BGN, Macro_ECM, Macro_VWF and Fib_CD74.

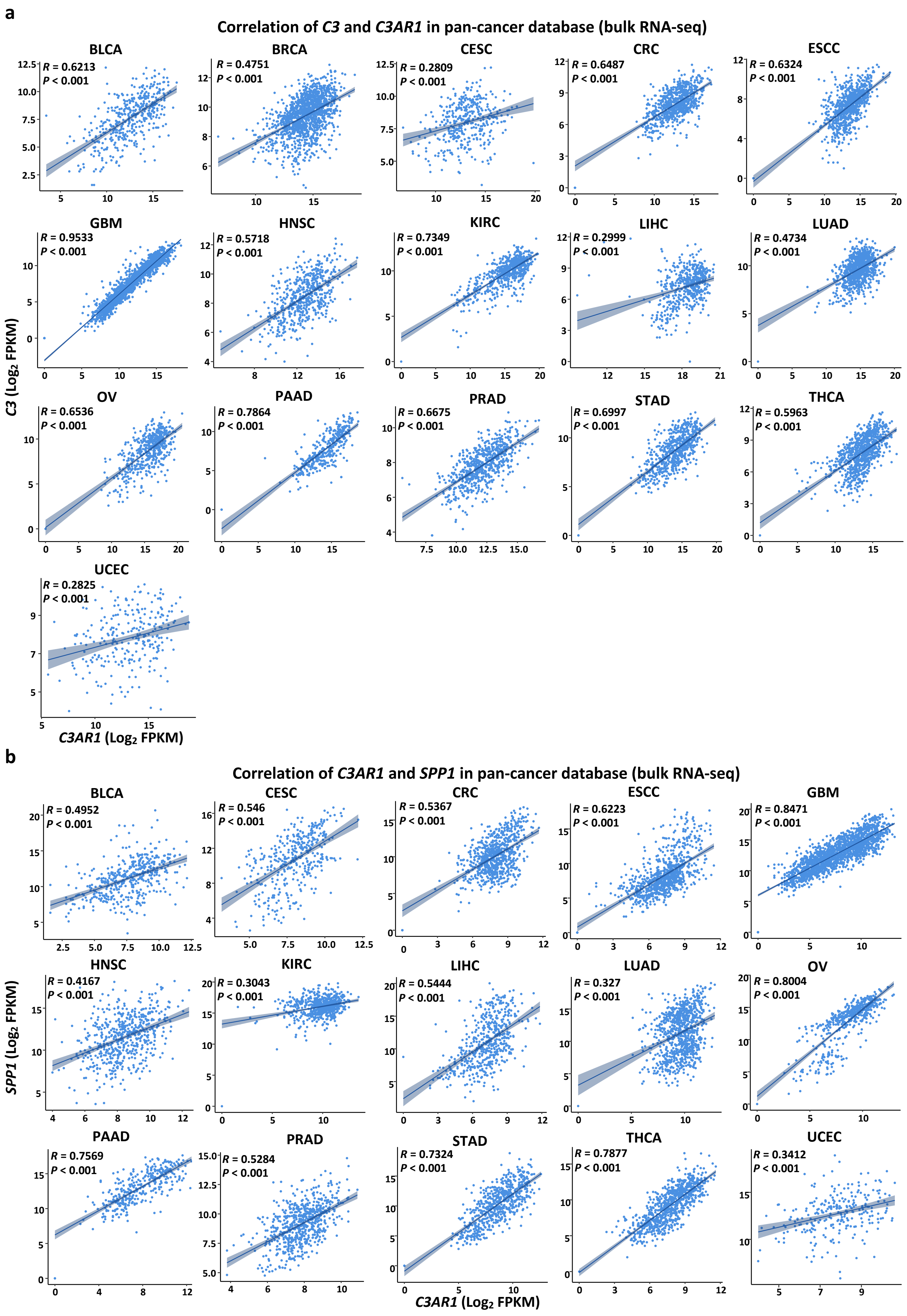

a
Correlation of C3 and C3AR1 in pan-cancer database (bulk RNA-seq)
BLCA R = 0.6213 P < 0.001
BRCA R = 0.4751 P < 0.001
CESC R = 0.2809 P < 0.001
CRC R = 0.6487 P < 0.001
ESCC R = 0.6324 P < 0.001
GBM R = 0.9533 P < 0.001
HNSC R = 0.5718 P < 0.001
KIRC R = 0.7349 P < 0.001
LIHC R = 0.2999 P < 0.001
LUAD R = 0.4734 P < 0.001
OV R = 0.6536 P < 0.001
PAAD R = 0.7864 P < 0.001
PRAD R = 0.6675 P < 0.001
STAD R = 0.6997 P < 0.001
THCA R = 0.5963 P < 0.001
UCEC R = 0.2825 P < 0.001
C3 (Log2 FPKM)
C3AR1 (Log2 FPKM)
b
Correlation of C3AR1 and SPP1 in pan-cancer database (bulk RNA-seq)
BLCA R = 0.4952 P < 0.001
CESC R = 0.546 P < 0.001
CRC R = 0.5367 P < 0.001
ESCC R = 0.6223 P < 0.001
GBM R = 0.8471 P < 0.001
HNSC R = 0.4167 P < 0.001
KIRC R = 0.3043 P < 0.001
LIHC R = 0.5444 P < 0.001
LUAD R = 0.327 P < 0.001
OV R = 0.8004 P < 0.001
PAAD R = 0.7569 P < 0.001
PRAD R = 0.5284 P < 0.001
STAD R = 0.7324 P < 0.001
THCA R = 0.7877 P < 0.001
UCEC R = 0.3412 P < 0.001
SPP1 (Log2 FPKM)
C3AR1 (Log2 FPKM)

Supplementary Fig. S15. Pan-cancer correlation of key interaction proteins, related to Fig. 7. **a** Correlation between *C3* and *C3AR1* gene expression at a pan-cancer level. **b** Correlation between *SPP1* and *C3AR1* gene expression at a pan-cancer level.

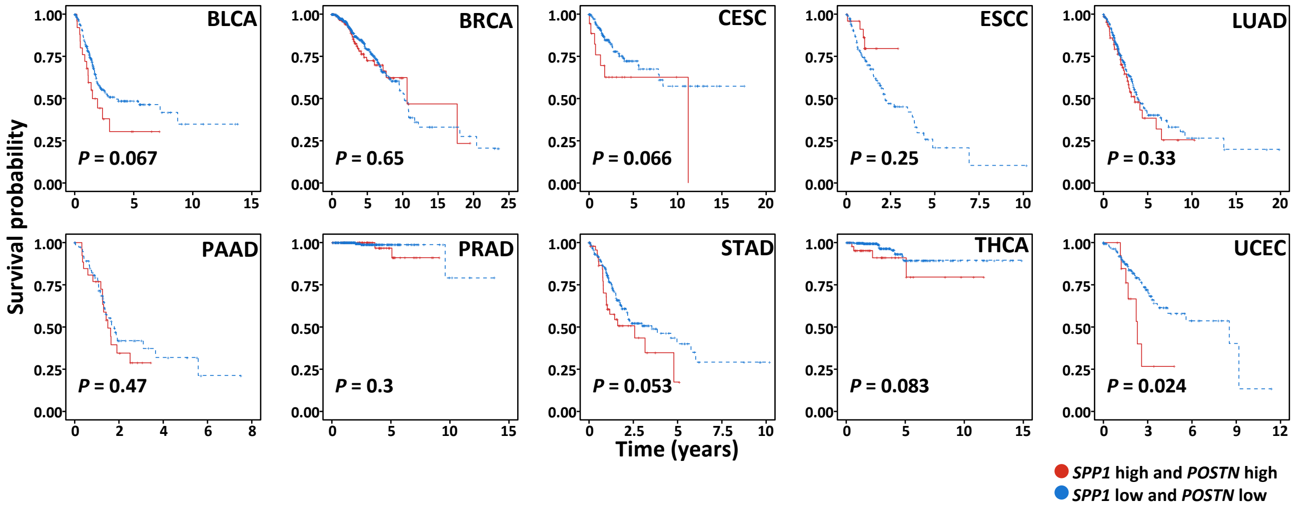

Supplementary Fig. S16. Prognostic correlation of *SPP1* and *POSTN* co-expression, related to Fig. 7.

Kaplan–Meier curves showing the association between co-expression of *SPP1* and *POSTN* and prognosis, with *P*-value calculated using the log-rank test.

Supplementary Table legends

Supplementary Table S1. Sample information of public single cell RNA-seq data.

Supplementary Table S2. Sample information of public spatial transcriptomics data.

Supplementary Table S3. Sample information of public bulk RNA-seq data.

Supplementary Table S4. Bulk RNA-seq survival data (TCGA).

Supplementary Table S5. Bulk RNA-seq tumor mutation burden (TMB) data (TCGA).

Supplementary Table S6. The differentially expressed genes in each Macrophages subtype.

Supplementary Table S7. M1 - M2 marker genes.

Supplementary Table S8. Bulk RNA-seq GSVA result.

Supplementary Table S9. Bulk RNA-seq xCell result.

Supplementary Table S10. Immunotherapy Sample information of public bulk RNA-seq data.

Supplementary Table S11. Mutation-associated neoantigen (MANA) marker genes.

Supplementary Table S12. SCPA metabolic pathways.
